# Recent Progress on Engineering Highly Efficient Porous Semiconductor Photocatalysts Derived from Metal–Organic Frameworks

**DOI:** 10.1007/s40820-018-0235-z

**Published:** 2019-01-08

**Authors:** Wenwen Zhan, Liming Sun, Xiguang Han

**Affiliations:** Jiangsu Key Laboratory of Green Synthetic Chemistry for Functional Materials, Department of Chemistry, School of Chemistry and Chemical Engineering, Jiangsu Normal University, Xuzhou, 221116 People’s Republic of China

**Keywords:** Metal–organic frameworks, Derivatives, Porous structure, Photocatalysis

## Abstract

Porous structures offer highly accessible surfaces and rich pores, which facilitate the exposure of numerous active sites for photocatalytic reactions, leading to excellent performances. Recently, metal–organic frameworks (MOFs) have been considered ideal precursors for well-designed semiconductors with porous structures and/or heterostructures, which have shown enhanced photocatalytic activities. In this review, we summarize the recent development of porous structures, such as metal oxides and metal sulfides, and their heterostructures, derived from MOF-based materials as catalysts for various light-driven energy-/environment-related reactions, including water splitting, CO_2_ reduction, organic redox reaction, and pollution degradation. A summary and outlook section is also included.

## Highlights

- In this review, we survey the recent developments in the fabrication of metal–organic framework (MOF)-derived porous semiconductor photocatalysts toward four kinds of energy-/environment-related reactions.
- A comprehensive summary of highly efficient MOF-derived photocatalysts, particularly porous metal oxides and metal sulfides, and their heterostructures are provided.
- Enhanced photocatalytic performance achieved with MOF-derived porous heterostructures as the photocatalyst is discussed in detail.

## Graphical abstract

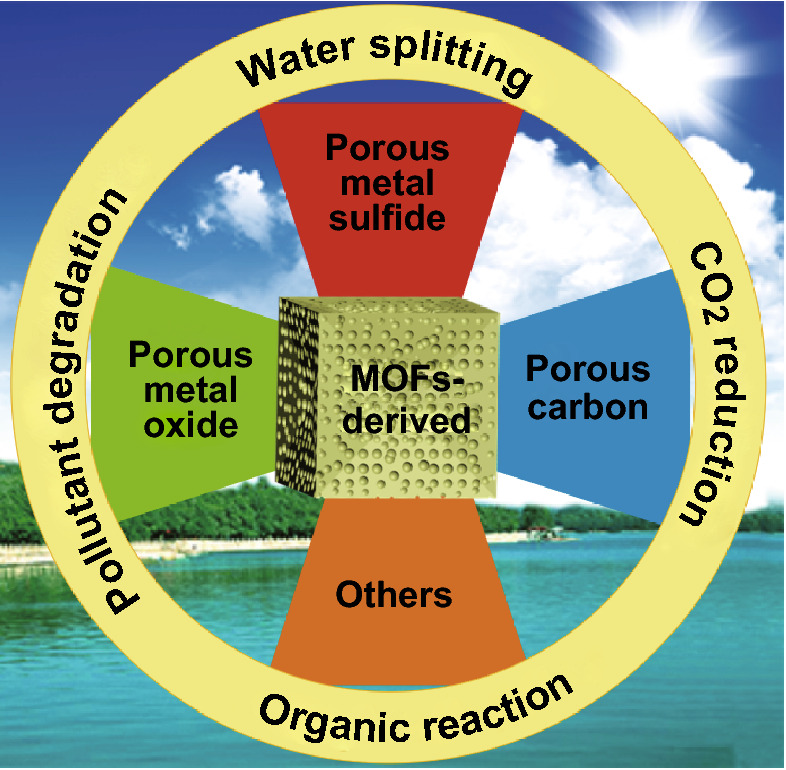

## Introduction

Global energy and environment issues have attracted much attention. Photocatalytic chemical processes, including hydrogen (H_2_) generation from water, carbon dioxide (CO_2_) reduction, pollution degradation, and organic chemical reactions, can convert solar energy to chemical energy, making them very promising in solving the energy and environment issues in a sustainable and environmentally friendly way [1–4]. Since the discovery of TiO_2_ as a photocatalyst for H_2_ production from water, a lot of efforts have been devoted to developing highly efficient semiconductor-based photocatalysts [5–13]. To date, metal oxides such as ZnO [14–16] and TiO_2_ [17–21], metal sulfides like CdS [22–24], carbon materials, for example, g-C_3_N_4_ [25, 26], and their heterostructures have shown great performances in photocatalysis. However, defects in low light utilization efficiency, improper band position, fast recombination of charge carriers, and photocorrosion have accelerated the investigation on strategies to close the gaps and design more efficient photocatalysts.

Porous micro–nanostructures can offer highly accessible surfaces and rich pores, which favor the exposure of numerous active sites in reactions, shorten the transfer distance to the pore surface for photoexcited carriers, and provide unrestricted diffusion of substrates and products, leading to their excellent performances in photocatalysis [27–33]. In addition, fabrication of semiconductor-based heterojunctions, including semiconductor–metal heterojunction and semiconductor–semiconductor heterojunction, is another useful strategy for enhancing the photocatalytic activity [10, 34–42]. Proper heterojunctions can tune the band gap, encourage the separation and migration of photogenerated electron–hole pairs, and enhance the efficiency of light utilization. In the last few years, much progress has been made in the design of photocatalysts with porous structures and heterostructures; however, the rational design of photocatalysts is hard because of complicated processes in traditional synthesis. Therefore, efficient and easily preparable photocatalysts with beneficial structural features are desired.

Recently, MOFs, well known as porous coordination polymers consisting of metal nodes and organic ligands, have attracted much attention and shown great potential for various applications, including photocatalysis, due to their fine-tuned structures, high specific surface areas, controlled pore structures, and various components [43–49]. Moreover, via the well-designed modification of MOF-based materials, not only the reactant adsorption and light absorption but also the charge separation and reactant activation can be largely promoted, leading to enhanced photocatalytic performances [50–53]. However, the poor stability and poor electronic conductivity of MOFs hinder their usage in the photocatalytic field. As an alternative, recent studies have found that MOFs can serve as precursors for porous semiconductor materials, including porous metal oxides, carbon materials, and metal sulfides, and their heterostructures, through the facile thermal treatment or sulfidation process [54–60]. Via the controlled derivation of MOFs in certain conditions, the as-synthesized products can maintain some of the initial structural features of parent MOFs or promote electrical conductivity, while maintaining the open diffusion channels and ensuring the monodispersion of metal centers, making them very promising in photocatalysis. More interestingly, porous heterostructures or solid solutions can be rationally derived from MOF-based hybrids or multimetallic MOFs, and the procedure has the following merits: (1) the versatility of MOFs in metal nodes and ligands endows enough choices for fabricating heterostructures or solid solutions, and the band gap of the obtained derivatives can be easily tuned by altering the metal or component ratios; (2) the in situ synthesis with MOFs as the precursor prevents the poor structural stability and weak coupling between the individual components of a heterostructure and solid solution; and (3) the in situ uniform distribution of metal nodes in multimetallic MOF precursors at the molecular level can contribute to an increase in the active sites, which can effectively participate in photocatalytic reactions. Therefore, MOFs can act as ideal precursors for rationally designed photocatalysts with enhanced performances.

**Fig. 1:**
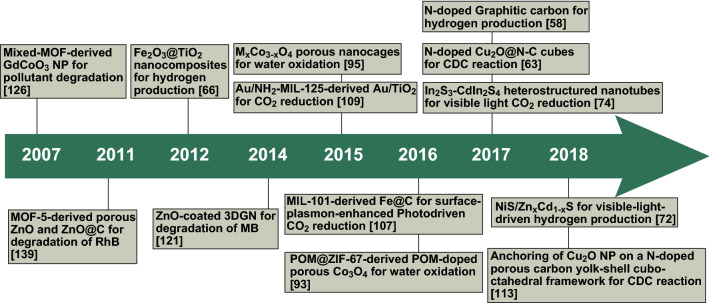
Timeline of the important breakthroughs in the fabrication of MOF-derived photocatalysts for various reactions

In the past few years, a lot of progress has been made on porous photocatalysts, including porous metal oxides, porous metal sulfides, and porous carbon, and their heterostructures, derived from monometallic MOFs, multimetallic MOFs, or MOF-based hybrids for water-splitting reactions, pollutant degradation, CO_2_ reduction, and organic synthesis. A timeline showing the breakthrough in the fabrication of highly efficient MOF-derived photocatalysts is shown in Fig. 1. With features of a porous structure and/or heterostructure, which are beneficial for increasing light utilization efficiency and promoting the separation and migration of photoinduced electron–hole pairs, these MOF-derived photocatalysts have acted well in enhancing photocatalytic performances. However, a specialized discussion on the progress achieved in the photocatalytic application of MOF-derived porous materials is very rare. Herein, we endeavor to give a comprehensive summary of the progress in four parts, i.e., (1) photocatalytic water splitting, including the photocatalytic hydrogen evolution reaction and oxygen evolution reaction; (2) photocatalytic degradation of pollutants, particularly dye pollutants; (3) photocatalytic CO_2_ reduction to CO or hydrocarbons; and (4) photocatalytic organic reactions. A brief summary and outlook are included in the final section.

## General Methods

Firstly, we give a brief summary of the synthetic strategies used to fabricate MOF-derived photocatalysts, including porous metal oxides, porous metal sulfides, and carbon materials. Depending on the different types of MOF-derived photocatalysts, the synthetic strategies can be summarized, as given in Table 1, which includes the corresponding examples.

**Table 1:** Brief summary of general methods for the fabrication of MOF-derived photocatalysts

MOFs-derived photocatalysts	General methods	Examples
Porous metal oxides; porous metal oxides doped with C or N	Direct calcination of the corresponding MOFs under different atmospheres	Porous ZnO [61], porous C-doped ZnO [62], and N-doped Cu_2_O@N–C [63]
Porous metal oxide-based heterostructures	Direct calcination of heterometallic MOFs under different atmospheres	ZnO/NiO porous hollow spheres [64] and ZnO/Co_3_O_4_ [65]
	Calcination of MOF-based heterostructure	Fe_2_O_3_@TiO_2_ [66], ZnO/Au [67], and porous Co_3_O_4_/CuO [68]
Porous metal sulfides	Direct sulfidation of MOFs	Yolk–shell CdS [69]
	Sulfidation of MOFs’ derivative	Co_4_S_3_ [70]
Porous metal sulfides-based heterostructure	Sulfidation of heterometallic MOFs or their derivatives	Hollow Co-based bimetallic sulfide [71] and NiS/Zn_*x*_Cd_1−*x*_S [72]
	Modification of MOFs’ derivatives	CdS/ZCO [73], and In_2_S_3_–CdIn_2_S_4_ nanotubes [74]
Porous carbon materials	Direct calcination of MOFs	N-doped graphitic carbon [58]

## Photocatalytic Water Splitting

Hydrogen, which can be renewably produced from a variety of (non-fossil) feedstocks, is a globally accepted clean energy carrier. Water splitting, which involves two half-reactions, i.e., hydrogen evolution reaction (HER) and oxygen evolution reaction (OER), has been considered as an attractive route to sustainable H_2_ generation [75]. Photocatalytic water splitting into H_2_ and O_2_ is a typical uphill reaction with a positive Gibbs energy change (Δ*G* = + 237.13 kJ mol^−1^), which requires a photocatalyst to trigger the reaction and convert solar energy to storable hydrogen energy [76]. Thermodynamically, the conduction band (CB) of the photocatalyst must be located more negative than the H^+^/H_2_ energy level (− 0.41 eV vs NHE at pH 7) and the valence band (VB) of the photocatalyst must be located more positive than the O_2_/H_2_O energy level (+ 0.82 eV vs NHE at pH 7) [77]. However, the overall water splitting requires higher photon energy than 1.23 eV due to the large overpotential caused by the charge transfer process and interaction between catalysts, reactants, and products. Therefore, the development of an overall water-splitting system remains a great challenge, and photocatalytic water splitting is generally studied separately. To date, many efficient porous photocatalysts derived from MOFs have been developed for HER; however, more progress needs to be made on OER (Table 2).

**Table 2:** Selected MOF derivatives that serve as photocatalysts for HER and OER

Photocatalyst	MOF precursors	*E*_g_ (eV)	Target reaction	Illumination range	Sacrificial reagent	Production rate [µmol (g h)^−1^]^a^/TOF (s^−1^)^b^	Recycled times	References
Co-Zn_0.5_Cd_0.5_S	ZnCo–ZIF	2.45	HER	Visible light (> 420 nm)	Na_2_S–Na_2_SO_3_	17,360	6	[90]
NiS/Zn_0.5_Cd_0.5_S	Ni/ZnCd–MOF	2.32	HER	Visible light (> 420 nm)	Na_2_S–Na_2_SO_3_	16,780	5	[72]
Zn_0.5_Cd_0.5_S	ZIF-8	–	HER	Visible light (> 420 nm)	Na_2_S–Na_2_SO_3_	12,130	6	[90]
CdS/ZCO	ZnCo–ZIF	2.1	HER	Visible light (> 420 nm)	Lactic acid	3978.6	4	[73]
Yolk–shell CdS	Cd–Fe–PBA	2.24	HER	Visible light	Na_2_S–Na_2_SO_3_	3051.4	4	[69]
Hollow Fe_2_O_3_–TiO_2_–PtO_*x*_	MIL-88B@TiO_2_	–	HER	Visible light (> 420 nm)	Lactic acid	1100	5	[82]
HP-CdS	MIL-53(Al)	~ 2.4	HER	Visible light (> 380 nm)	Na_2_S–Na_2_SO_3_	634	4	[88]
Fe_2_O_3_@TiO_2_	MIL-101@ TiO_2_	–	HER	Visible light (> 420 nm)	TEA	~ 625	3	[66]
FeO_*x*_–carbonaceous composites	MIL-88B/rGO	–	HER	Visible light (> 420 nm)	TEA	264.1	4	[85]
ZnO/Au	Au/ZIF-8	3.17	HER	Visible light (> 400 nm)	Na_2_S–Na_2_SO_3_	29.8	4	[67]
PHIC	In-MIL-68	–	HER	UV–Vis light	TEOA	2,700,000	5	[86]
Hollow Cu-TiO_2_/C/Pt	SiO_2_@MOF-199/Ti	2.89	HER	UV–Vis light	Ethanol	14,049	3	[91]
Co_4_S_3_/CdS	Co–MOF	2.0	HER	UV–Vis light	Lactic acid	12,360	5	[70]
Pt–Zn_3_P_2_–CoP	ZnCo–ZIF	–	HER	UV–Vis light	Methanol	9150	5	[65]
Pt–ZnS–CoS	ZnCo–ZIF	–	HER	UV–Vis light	Methanol	8210	5	[84]
Pt–ZnO–Co_3_O_4_	ZnCo–ZIF	–	HER	UV–Vis light	Methanol	4450	5	[83]
Co_3_O_4_/TiO_2_	Ti/Co-PA	–	HER	UV–Vis light	Methanol	~ 7000	–	[84]
Pd/TiO_2_	NH_2_-MIL-125	~ 3.2	HER	UV–Vis light	Methanol	979.7	3	[83]
Cu/TiO_2_–AA	MOF-199@ TiO_2_	–	HER	UV light	Methanol	62.16	–	[81]
N-doped graphitic carbon/Pt	ZIF-8	–	HER	UV–Vis light	TEOA	18.5	–	[58]
N-doped graphitic carbon	ZIF-8	–	HER	UV–Vis light	TEOA	5	–	[58]
700-CoO_*x*_–C	ZIF-67	–	OER	Visible light (> 420 nm)	[Ru(bpy)_3_]^2+^–Na_2_S_2_O_8_	0.039	3	[92]
Co_3_O_4_/CuO-3 HPNCs	ZIF-67/Cu HD	–	OER	Visible light (> 420 nm)	[Ru(bpy)_3_]^2+^–Na_2_S_2_O_8_	4.9 × 10^−3^	5	[68]
POM-doped porous Co_3_O_4_	PW_12_@ZIF-67	–	OER	Visible light (> 420 nm)	[Ru(bpy)_3_]^2+^–Na_2_S_2_O_8_	1.11 × 10^−3^	3	[93]
Co_*x*_Fe_3−*x*_O_4_	PBA	–	OER	Visible light (> 420 nm)	[Ru(bpy)_3_]^2+^–Na_2_S_2_O_8_	5.4 × 10^−4^	4	[94]
Co_3_O_4_ nanocages	PBA	–	OER	Visible light (> 420 nm)	[Ru(bpy)_3_]^2+^–Na_2_S_2_O_8_	3.2 × 10^−4^	–	[95]
C,N-doped ZnO	ZIF-8	2.98	OER	Solar-simulated light	AgNO_3_	–	–	[96]

^a^Production rate unit of the listed photocatalytic HER

^b^Production rate unit of the listed photocatalytic OER

### Photocatalytic HER

In the hydrogen evolution half-reaction system, the other half-reaction is replaced by the oxidation of an appropriate sacrificial reductant, such as methanol, ethanol, triethanolamine, triethylamine, ascorbic acid, lactic acid, and Na_2_S/Na_2_SO_3_ pairs [77]. Similar to that for other photocatalytic systems, the design principles for the HER photocatalysts involve a suitable band gap, enhanced charge transfer efficiency, and numerous active sites. MOFs serve as ideal precursors for highly efficient HER photocatalysts because of their ability to provide various metal ions (as options), facilitate the doping of C or N to MOF-derived semiconductors, and give porous structures with high surface areas.

#### MOF-Derived Porous Metal Oxides

**Fig. 2:**
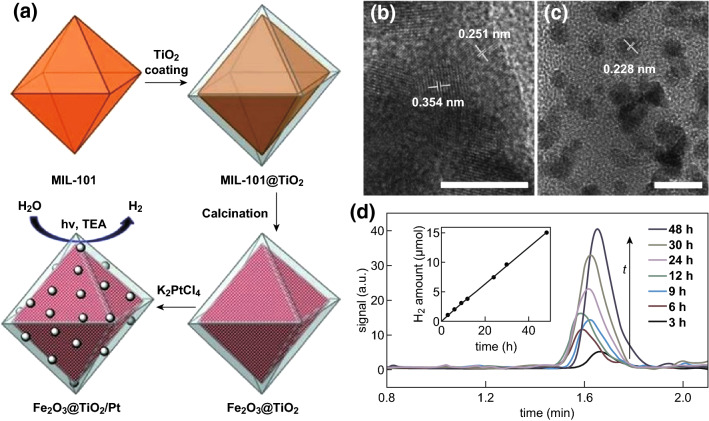
**a** Schematic illustration of MOF-templated synthesis of Fe_2_O_3_@TiO_2_ and its use for photocatalytic hydrogen production after deposition of Pt particles; high-resolution TEM images of Fe_2_O_3_@TiO_2_, both **b** as-synthesized and **c** after Pt particles have been deposited. The scale bars represent 10 nm; **d** H_2_ produced by Fe_2_O_3_@TiO_2_ at various times over 48 h under visible-light illumination. The inset shows the amount of H_2_ produced over this period. Reprinted with permission from Ref. [66]

Metal oxide nanostructures, one of the most important semiconductor nanomaterials, have attracted much attention as photocatalysts for water splitting. TiO_2_ has been considered the most interesting photocatalyst for water splitting, especially for HER, due to its suitable band positions, low cost, low toxicity, high stability, and n-type semiconducting nature [78, 79]. However, highly efficient TiO_2_-based photocatalysts need to be developed because factors such as imperfect light absorption range and quick recombination of photoinduced carriers limit the use of pure TiO_2_. Several studies indicated that fabricating TiO_2_-based heterojunctions with metals or other semiconducting materials led to extended light absorption to the visible-light range, as well as suppression of the recombination of photoinduced carriers, leading to higher photocatalytic activity [80]. With MOFs as templates or precursors, facile design of TiO_2_-based heterostructures can be achieved. For example, Lin’s group developed a two-step approach to fabricate Fe_2_O_3_@TiO_2_ nanostructures with MIL-101(Fe) as the precursor. First, the MIL-101(Fe)@amorphous TiO_2_ precursor was obtained by coating MIL-101 nanoparticles with TiO_2_ (shell) via acid-catalyzed hydrolysis and condensation of titanium(IV) bis(ammonium lactato)dihydroxide (TALH) in water; then, the precursor was calcined in air to obtain Fe_2_O_3_@TiO_2_ nanostructures (Fig. 2) [66]. This heterostructure photocatalyst exhibited interesting properties and enabled visible-light-driven hydrogen production from water, while neither of the individual components had such ability. Xiong’s group has also reported a series of hollow TiO_2_-based photocatalyst derived from the MOF@TiO_2_ core–shell precursor for H_2_ evolution from water [81]. Compared with products obtained via other calcination approaches, Cu/TiO_2_–AA, the product obtained by simultaneous etching and reduction with ascorbic acid (AA), can better preserve the octahedral-shaped shells and crystal phase as well as prevent the formation of carbon residues and cocatalyst aggregation, leading to improved efficiency in photocatalysis. These reports indicate that mixing TiO_2_ with suitable nanomaterials can offer a solution to enhance its photocatalytic activity because of the synergistic effect.

Accordingly, other MOF-derived TiO_2_-based heterostructures have been obtained. For instance, the hollow hybrid Fe_2_O_3_–TiO_2_–PtO_*x*_ photocatalyst was fabricated with nanosized MIL-88B(Fe)-based heterostructures as a hard template [82]. Because of the presence of two cocatalysts on opposite sides, the Fe_2_O_3_–TiO_2_–PtO_*x*_ hollow photocatalyst showed high activity toward visible-light-induced H_2_ generation with a high production rate of 1100 µmol (g h)^−1^, which might be attributed to Fe doping on TiO_2_, separation of PtO_*x*_ and α-Fe_2_O_3_ nanoparticles as cocatalysts, as well as the short migration distance of electrons and holes to the surface. Besides, combining TiO_2_ with noble metal materials, Pd as an example, could be easily prepared with MOF-derived hierarchical TiO_2_ as support and photoreduction agent at the same time [83]. With a Pd loading amount of 1.5%, the Pd/TiO_2_ photocatalyst showed optimized rates of H_2_ evolution of ~ 2449 and ~ 281.7 µmol (g h)^−1^ under UV–Vis light and simulated solar light, respectively.

As it is an n-type semiconductor, TiO_2_ can be combined with p-type semiconductors to fabricate p–n heterojunctions with enhanced photocatalytic performance due to more effective charge separation; rapid charge transfer to the surface of catalyst; longer lifetime of the charge carriers; and separation of locally incompatible reduction and oxidation reactions in nanospace [40, 41]. The versatile metal nodes of MOFs facilitate the facile and rational design of TiO_2_-based p–n heterojunctions. For example, Mondal et al. reported an improved Co_3_O_4_/TiO_2_ photocatalytic system with p–n heterojunction derived from several newly developed Co–MOFs [84]. As mentioned, the obtained p–n heterojunction consisted of spinel Co_3_O_4_ and anatase TiO_2_. With an optimized Co loading of 2 wt%, the Co_3_O_4_/TiO_2_ could deliver hydrogen at a high rate of 7000 µmol (g h)^−1^ under UV–Vis light due to the synergistic effect of the formed small heterojunction and cocatalytic role of Co_3_O_4_, which facilitated interfacial charge transfer and electron–hole separation.

**Fig. 3:**
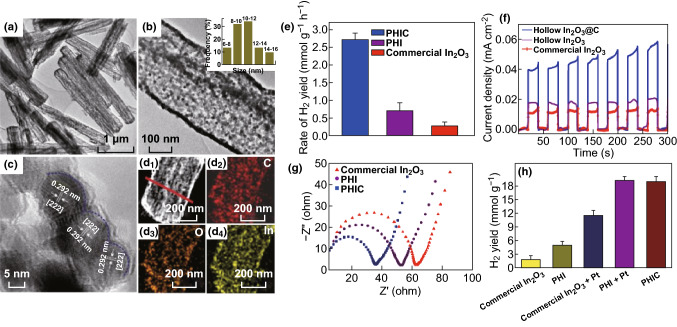
**a**–**c** TEM and HRTEM images of PHIC. **d** STEM image and EDXS elemental mapping of C, O, and In. **e** mass-normalized H_2_ yield rates over PHIC, PHI, and commercial In_2_O_3_ under sunlight illumination. **f** photocurrent densities of PHIC, PHI, and commercial In_2_O_3_ under non-illuminated (i.e., dark) and illuminated (i.e., light) conditions. **g** Electrochemical impedance spectroscopy Nyquist plots of PHIC, PHI, and commercial In_2_O_3_. **h** mass-normalized H_2_ yield for 7 h over commercial In_2_O_3_, PHI, commercial In_2_O_3_ with Pt, PHI with Pt, and PHIC without Pt under simulated sunlight illumination. Reprinted with permission from Ref. [86]

Other metal oxide (In_2_O_3_, ZnO, Fe_2_O_3_, etc.)-based porous photocatalysts with high activities for hydrogen evolution have also been easily prepared with MOFs as templates and/or precursors [67, 85–87]. For instance, an In_2_O_3_-based photocatalyst, namely PHIC, with highly improved activity for hydrogen evolution was fabricated via a facile thermal decomposition of In-MIL-68 template by our group (Fig. 3) [86]. This PHIC catalyst with a hollow hexagonal micro-rod shape was assembled using In_2_O_3_@carbon core–shell nanoparticles. Due to the synergistic effect of efficient separation of photogenerated electron–hole pairs caused by the carbon coating, enhanced optical absorption attributed to hollow characters, and improved accessibility rendered by the porous structure, PHIC could exhibit a photocatalytic activity comparable to that of the Pt/In_2_O_3_ photocatalyst toward hydrogen evolution with an extremely high production rate of 2,700,000 µmol (g h)^−1^ under solar-simulated light. In addition, ZnO, a typical semiconductor with absorption in the UV region, has been optimized as a better photocatalyst by compounding it with other materials, such as noble metal nanoparticles (NPs) and other metal oxides. With Zn–MOF/metal NP hybrids as the precursor, ZnO/metal NP heteromaterials with porous structures could be easily obtained [67]. Wang group fabricated the Au/ZnO NP photocatalyst with yellow fluorescent GSH-Au nanoclusters (NCs)/ZIF-8 NPs as the precursor, and this photocatalyst could extend the absorption of ZnO to the visible-light region. The obtained An/ZnO NP photocatalyst could achieve a hydrogen generation rate of ~ 29 µmol (g h)^−1^ under visible-light illumination.

#### MOF-Derived Porous Metal Sulfides

**Fig. 4:**
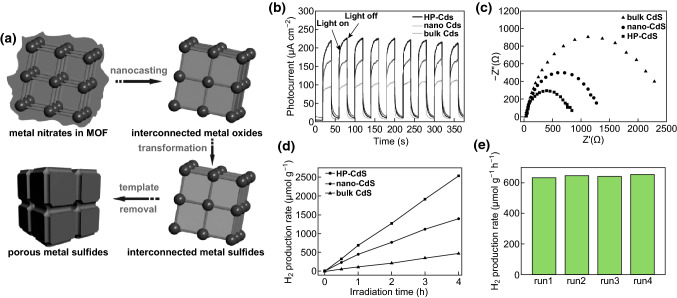
**a** Schematic illustration of the synthesis of hierarchically porous metal oxides/sulfides templated by MOFs by a nanocasting process. **b** Photocurrent response and **c** EIS Nyquist plots for bulk CdS, nano-CdS as well as HP-CdS. **d** Comparison of the photocatalytic hydrogen production rates of bulk CdS, nano-CdS, and HP-CdS. **e** Catalytic recyclability of HP-CdS in hydrogen production by water splitting. Reprinted with permission from Ref. [88]

Cadmium sulfide (CdS) is another promising photocatalyst for solar-driven hydrogen evolution due to its visible-light response (*E*_g_ = 2.4 eV) and conduction band located at a suitable energy level. However, there are two drawbacks for the CdS photocatalyst, i.e., easy recombination of photogenerated hole–electron pairs and high photocorrosion in aqueous media, which restrict its practical application. In order to enhance the photocatalytic activity of CdS, various strategies have been introduced, including increasing specific surface area and modifying CdS with cocatalysts or incorporating other materials to form solid solutions, each of which could be handily achieved with the MOF-derivation strategy. Notably, CdS materials with high surface areas as photocatalysts may still suffer from poor stability, but they can demonstrate improved activities by promoting the separation of photoinduced hole–electron pairs. For example, Xiao and Jiang synthesized a hierarchically porous CdS (HP-CdS) utilizing thermally stable MOFs as hard templates to afford porous CdO, and subsequently, CdS by a nanocasting method (Fig. 4) [88]. The obtained HP-CdS showed a BET surface area of 119 m^2^ g^−1^, which facilitated the effective inhibition of photogenerated electron–hole recombination, leading to a higher photocatalytic activity for HER than nano-CdS and bulk CdS under visible light (> 380 nm). In addition, CdS with complex hollow nanostructures, which provides multiple structural advantages for the photocatalytic reaction, such as enhanced light absorption, improved separation efficiency of the photoinduced charge carriers, and increased specific surface area, could be obtained with MOFs as templates or precursors. Wang and Liu et al. have reported the formation of a yolk–shell-structured CdS material by a two-step MOF-based approach, involving facile synthesis of uniform Cd–Fe-PBA micro-cubes and subsequent chemical sulfidation [69]. Due to the structural merits, including a 3D open structure, small size of primary nanoparticles, high specific surface area, and good structural robustness, the yolk–shell-structured CdS material could generate H_2_ from water under visible-light illumination with an excellent rate of 3051.4 µmol (g h)^−1^.

**Fig. 5:**
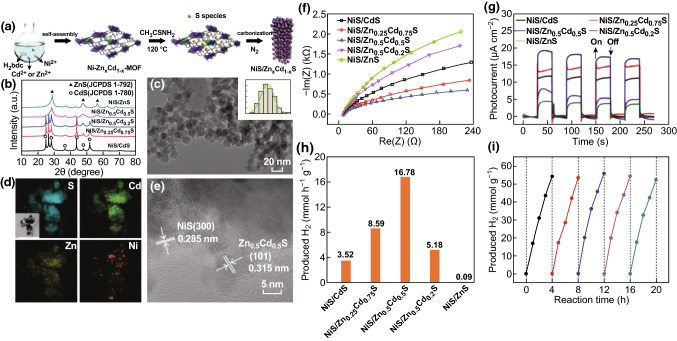
**a** Schematic illustration of the synthetic procedure for NiS/Zn_*x*_Cd_1−*x*_S. **b** PXRD patterns of NiS/Zn_*x*_Cd_1−*x*_S. **c** TEM image and particle size distribution (Fig. 2c inset) of NiS/Zn_0.5_Cd_0.5_S. **d** EELS elemental mapping images and **e** HRTEM image of NiS/Zn_0.5_Cd_0.5_S. **f** EIS plots, **g** photocurrent–time dependence, and **h** comparison of photocatalytic HER rates of NiS/Zn_*x*_Cd_1−*x*_S; **i** HER cycling test for NiS/Zn_0.5_Cd_0.5_S under visible-light irradiation. Reprinted with permission from Ref. [72]

MOF-based strategies for decorating CdS either with cocatalysts, or by incorporating other materials to form a solid solution, have attracted much attention due to the obvious merits of MOFs, such as variable composition and porosity. For example, CdS/Zn_*x*_Co_3−*x*_O_4_ (CdS/ZCO) hollow composites with high photocatalytic activity for visible-light-induced H_2_ generation from water have been fabricated by decorating CdS nanoparticles on Zn/Co–ZIF derivatives (Zn_*x*_Co_3−*x*_O_4_) [73]. With an optimized loading content of CdS (30%) on the ZCO surface, CdS/ZCO achieved a high H_2_ production rate of about 3978.6 μmol (g h)^−1^, which was attributed to the synergistic effect, i.e., the efficient charge separation and transfer between the phase boundary of CdS and ZCO. Similarly, Co–MOF-derived onion slice-type hollow-structured Co_4_S_3_ was developed and decorated with CdS nanoparticles for photocatalytic hydrogen production by Kim’s group [70]. The optimized Co_4_S_3_/CdS material led to an enhanced rate of H_2_ generation of 12,360 μmol (g h)^−1^ under simulated solar light irradiation. The low density, hollow interior, and shell permeability of the onion-type composite helped in accelerating the charge separation and transfer in photocatalytic reactions. They also prepared MOF-derived Ni_2_P nanoparticles as the cocatalyst of CdS, and the Ni_2_P/CdS heterostructure exhibited great improvement in performance during photocatalytic HER due to the decreased rate of charge carrier recombination [89]. Moreover, a family of photocatalysts (NiS/Zn_*x*_Cd_1−*x*_S) for HER has been developed by decorating CdS with NiS as a cocatalyst and simultaneously incorporating Zn to form solid solutions [72]. As shown in Fig. 5, Cheng’s group utilized Zn- and Ni-doped Cd–MOFs as the sacrificial templates to form the NiS/Zn_*x*_Cd_1−*x*_S series via solvothermal sulfidation and thermal annealing. By adjusting the doping metal concentration in the MOFs, the chemical compositions and band gaps of the heterojunctions were fine-tuned, leading to an optimized HER rate of up to 16,780 μmol (g h)^−1^ with NiS/Zn_0.5_Cd_0.5_S as the photocatalyst under visible-light irradiation. In-depth DFT calculations revealed the importance of NiS in accelerating the water dissociation kinetics, which was crucial for photocatalytic HER. Notably, a visible-light catalytic system with a HER rate of up to 17,360 μmol (g h)^−1^ over a Co-Zn_0.5_Cd_0.5_S solid solution catalyst was developed via a sulfidation process of Co/Zn–ZIF in the presence of Cd^2+^ [90]. The doping of Co evenly around the skeleton of the porous Zn_0.5_Cd_0.5_S solid solution played an important role in improving the photocatalytic activity, as compared to that of Zn_0.5_Cd_0.5_S, which displayed a HER rate of 12,130 μmol (g h)^−1^ under the same reaction condition.

**Fig. 6:**
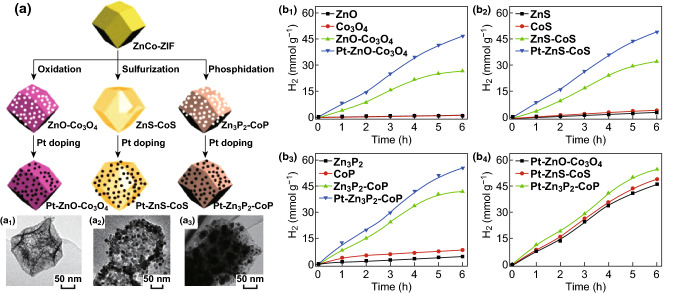
**a** Schematic illustration of the fabrication of Pt–ZnO–Co_3_O_4_, Pt–ZnS–CoS, and Pt–Zn_3_P_2_–CoP photocatalysts. **a**_**1**_**–a**_**3**_ TEM images of Pt–ZnO–Co_3_O_4_, Pt–ZnS–CoS, and Pt–Zn_3_P_2_–CoP. **b**_**1**_**–b**_**4**_ Photocatalytic hydrogen generation as the function of reaction time over different photocatalysts. Reprinted with permission from Ref. [65]

As per the reports mentioned above, utilizing MOFs as templates or precursors to construct heterojunctions between a semiconductor material and another semiconductor material or a noble metal material is an effect way to improve the performance of semiconductors in photocatalytic HER by water splitting. Accordingly, Li et al. reported a series of Zn/Co–ZIF derivative/Pt photocatalytic systems, which exhibited high performance toward the photocatalytic HER under UV–Vis light (shown in Fig. 6) [65]. The synthesis processes involved the oxidation, sulfurization, or phosphorization of ZnCo–ZIF, and the subsequent photochemical doping of Pt nanoparticles, leading to Pt–ZnO–Co_3_O_4_, Pt–ZnS–CoS, and Pt–Zn_3_P_2_–CoP, respectively. The porous framework skeleton of the ZnCo–ZIF derivatives significantly enhanced the light utilization and simultaneously afforded abundant exposed catalytic active sites; the suitable band matching and strong electron coupling in the heterojunctions facilitated efficient electron–hole separation and transportation; the distribution of Pt nanoparticles on the porous structure offered enough redox active sites. These beneficial features were revealed to be responsible for the highly enhanced performances of the Zn/Co–ZIF derivative/Pt photocatalysts. This bimetallic MOF-directed fabrication strategy reported in this paper provided a new perspective to construct synergetic photocatalysts with excellent photocatalytic performances for water-splitting applications.

#### MOF-Derived Porous Carbon Materials

**Fig. 7:**
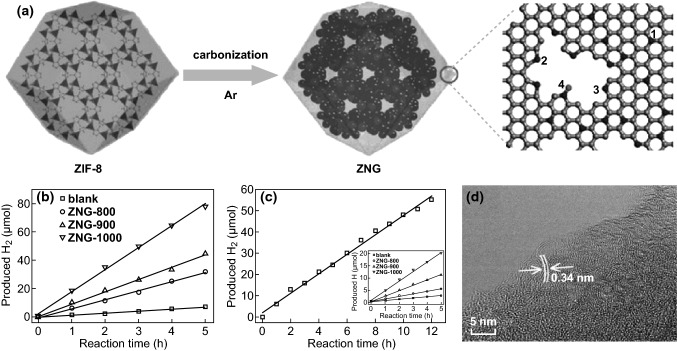
**a** Schematic illustration of the synthetic procedure of ZNGs and four types of bonding configurations of N atoms (1 graphitic N, 2 pyrrolic N, 3 pyridinic N, 4 pyridine-*N*-oxide). **b** Photocatalytic activities of ZNGs for H_2_ production with Pt as a cocatalyst. **c** Photocatalytic activity of ZNG-1000 under a prolonged light illumination for 12 h without Pt as a cocatalyst (inset: time course of the H_2_ evolution rate of ZNGs without Pt as a cocatalyst). **d** High-resolution TEM image of ZNG-1000. Reproduced with permission from Ref. [58]

In general, progress on the photocatalytic hydrogen evolution from water over MOFs-derived photocatalysts have been made mainly around metal-involving materials, like metal oxides, metal sulfides, or their composites, which are commonly used in the photocatalytic field. As alternatives, metal-free photocatalysts, such as carbon-based materials, demonstrating great performance are highly desired, due to their tunable molecular structures, abundance, and high chemical stability. Meanwhile, facile synthesis of metal-free photocatalysts is still highly challenging. Cheng and coworkers have developed a facile synthesis method for N-doped graphene carbon using a well-designed ZIF-8-template (Fig. 7) [58]. Under an Ar atmosphere at different temperatures, ZIF-8 were calcined to derive N-doped graphene (ZNG) analogs, which retained the polyhedron structure of the parent ZIF-8 particles and had nitrogen contents of 9–15 wt%; the contents of various N types in the materials were fine-tuned on the basis of the calcination temperatures. Among them, the product obtained at 1000 °C exhibited the best performance toward photocatalytic HER because of the highest content of graphitic nitrogen, which preserved the high mobility of the charge carriers and further affected the hydrogen evolution rate of the photocatalyst.

### Photocatalytic Water Oxidation

Water oxidation to O_2_ is regarded as the bottleneck of solar-driven water splitting, ascribed to the intrinsic difficulty in multiple-electron transfer and sluggish kinetics of the subsequent oxygen evolution [97, 98]. Although some noble metals and their oxides, such as IrO_2_ and RuO_2_, have attracted much attention as efficient catalysts for water oxidation, their high cost and low abundance have impeded their commercial utilization. Therefore, first-row transition metal oxides and their derivatives, including cobalt oxides, iron oxides, nickel oxides, and manganese oxides, have been explored as water oxidation catalysts due to advantages with regard to economy and stability [99–105]. In the water oxidation reaction system (i.e., OER), the reduction of sacrificial oxidants, such as AgNO_3_ and Na_2_S_2_O_8_, is normally used to replace the half-reaction of HER [77].

According to investigations, MOF-derived water oxidation photocatalysts, particularly cobalt oxide-based systems, are environmentally benign, thermally stable, inexpensive, and demonstrate high OER activity both in the electrochemical and in the photochemical fields. The Lu group developed a facile approach for the preparation of a porous cobalt oxide–carbon hybrid as a water oxidation catalyst by carbonizing nanocrystals of ZIF-67 in an inert atmosphere and subsequently air-calcining them [92]. Among the various CoO_*x*_/C hybrids obtained at different calcination temperatures, 700-CoO_*x*_/C acted as the best catalyst. The photocatalytic activity of 700–CoO_*x*_/C was accessed in the [Ru(bpy)_3_]^2+^–S_2_O_8_^2−^ system under visible light in a sodium phosphate buffer (pH 8.5) and a maximum turnover frequency (TOF) of up to 0.039 ± 0.03 s^−1^ per cobalt atom was estimated, which was among the highest TOFs for water oxidation with cobalt oxide-based photocatalysts.

**Fig. 8:**
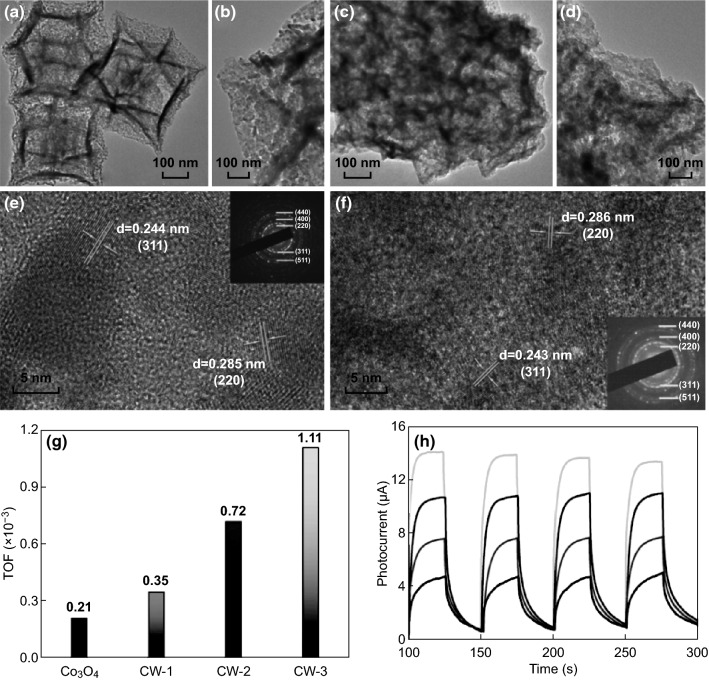
**a** Low- and **b** high-magnification TEM images of Co_3_O_4_ hollow dodecahedra. **c** Low- and **d** high-magnification TEM images of CW-2 hollow dodecahedra. **e** HRTEM image of Co_3_O_4_ hollow dodecahedra (inset is the SAED pattern recorded for the whole particle). **f** HRTEM image of CW-2 hollow dodecahedra (inset is the SAED pattern recorded from the whole particle). **g** TOF and **h** transient photocurrent response of Co_3_O_4_ and CW-*n* under visible-light irradiation (from left to right: Co_3_O_4_, CW-1, CW-2, and CW-3). Reprinted with permission from Ref. [93]

Furthermore, by combining ZIF-67 with suitable materials as precursors, adjustable cobalt oxide-based composites as photocatalysts for water oxidation could be fabricated. For example, the derivatives obtained by loading a single Keggin-type polyoxometalate (POM) cluster into each confined space of ZIF-67, i.e., POM@Co_3_O_4_ composites (CW-*n*, *n* depended on the added amount of POM) doped with highly dispersive molecular metal-oxo clusters, exhibited significantly improved photocatalytic activity in water oxidation compared to the pure MOF-derived nanostructure (as shown in Fig. 8) [93]. In the molecular cluster@oxide system, POMs accept and release electrons, thereby improving the separation of light-induced electrons and holes, which leads to higher catalytic activity with increasing POM concentration in the composite materials. Ding’s group synthesized a series of Co_3_O_4_/CuO hollow polyhedral nanocages (HPNC) using ZIF-67/Cu hydroxide (HD) polyhedrons with various Co/Cu molar ratios as sacrificial templates [68]. With an optimized Co/Cu molar ratio, Co_3_O_4_/CuO-3 HPNCs afforded a high TOF of 4.9 × 10^−3^ s^−1^ per metal atom and performed well in the stability test.

In addition, Prussian blue analogue (PBA), a type of crystalline MOF built from divalent and trivalent metal ions (such as Fe, Co, and Ni) linked by cyanide ligands, has also been chosen as the precursor to fabricate water oxidation photocatalysts, such as Fe/Co/Ni-oxides and their solid solutions. Ding’s group reported the use of low-cost porous iron-based oxides derived from the calcination of PBA (M_*x*_[Fe(CN)_6_]_y_, where M = Fe, Co, Ni) and porous Co-based oxide nanocages derived from the calcination of PBA (M_*x*_[Co(CN)_6_]_y_, where M = Fe, Co, and Mn) as catalysts in photocatalytic water oxidation [94, 95]. The obtained series of Fe photocatalysts followed the order of Co_*x*_Fe_3−*x*_O_4_ > Ni_*x*_Fe_3−*x*_O_4_ > Fe_2_O_3_, and Co_*x*_Fe_3−*x*_O_4_ afforded an initial TOF of 5.4 × 10^−4^ s^−1^ per transition metal atom. Meanwhile, the obtained series of Co photocatalysts followed the order of Co_3_O_4_ > Mn_*x*_Co_3−*x*_O_4_ > Fe_*x*_Co_3−*x*_O_4_, and Co_3_O_4_ porous nanocages exhibited the highest TOF of 3.2 × 10^−4^ s^−1^ per Co atom.

In summary, tremendous efforts have been devoted to develop efficient photocatalysts toward water splitting with MOFs or their hybrids as the precursors, and great progress has been made in this regard. However, the currently developed photocatalytic systems based on MOF-derived porous structures only involve one isolated half-reaction, and sacrificial reagents are needed to replace the other half-reaction. More studies have been performed on hydrogen evolution reaction than oxygen evolution reaction due to the intrinsic difficulty associated with OER in multiple-electron transfer and sluggish kinetics. However, the practical application of water splitting demands the production of stoichiometric amounts of H_2_ and O_2_ driven by sunlight without any sacrificial reagents. Therefore, photocatalytic overall water-splitting systems with high efficiency are highly desired. Moreover, the porous structures of photocatalysts can influence the attachment of gas bubbles on the surface of the catalysts, which can potentially block the active sites and prohibit mass transportation under strong gas evolution conditions, leading to bubble overpotential [106]. As far as we know, no investigation on the influence of pore structure of MOFs-derived photocatalysts on overpotential for water-splitting reaction, especially OER, has been performed, and therefore, further studies are needed.

## Photocatalytic CO_2_ Reduction

The global climate issues caused by the rapidly increasing CO_2_ emissions have accelerated studies on photocatalytic CO_2_ reduction to CO or hydrocarbons, which can not only decrease the CO_2_ level in the atmosphere, facilitating environmental protection, but also generate materials for chemical industry or energy storage. Nowadays, the photocatalytic CO_2_ reduction process is attracting growing attention because of its usage of solar power as the primary energy source. Photocatalytic CO_2_ reduction involves the following steps: light harvesting, separation, transfer of photogenerated charge carriers, as well as adsorption and conversion of CO_2_ [107]. Therefore, in addition to the impacts of light utilization, charge transfer efficiency, and active sites, the adsorption of CO_2_ is another important factor to expedite the CO_2_ reduction process in a photocatalytic system. The CO_2_ reduction products include HCOOH, CO, HCHO, CH_3_OH, and CH_4_. MOF-derived materials are considered highly potential photocatalysts for CO_2_ reduction due to their advantages such as structural and compositional variety and high surface areas. Some excellent works have been reported on MOF-derived materials as CO_2_ reduction photocatalysts (Table 3).

**Table 3:** Selected MOF derivatives that serve as photocatalysts for CO_2_ reduction

Photocatalyst	MOF precursors	*E*_g_ (eV)	Illumination range	Main products	Reaction rate (µmol (g h)^−1^)	Recycled times	References
Fe@C	MIL-101	–	UV–Vis light	CO	18,301	5	[108]
ZnMn_2_O_4_	Zn/Mn–MOF	2.51	UV–Vis light	CO	23.99	–	[111]
In_2_S_3_/CdIn_2_S_4_	MIL-68	2.21	Visible light	CO	825	6	[74]
C–Cu_2−*x*_S@g-C_3_N_4_	MOF-199	–	Visible light	CO	88.55	–	[109]
ZnO/NiO	Zn/Ni–MOF	–	UV–Vis light	CH_3_OH	1.57	–	[64]
Au/TiO_2_	Au/MIL-125	–	UV light	CH_4_	240^a^	2	[110]

^a^Unit: ppm (g h)^−1^

**Fig. 9:**
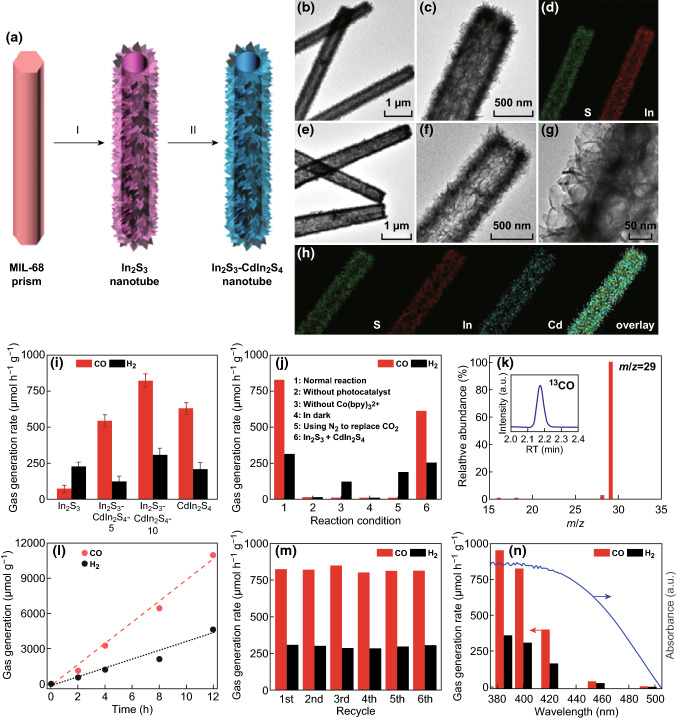
**a** Schematic illustration of the synthetic process of the hierarchical In_2_S_3_–CdIn_2_S_4_ heterostructured nanotube: (I) liquid phase sulfidation and (II) cation exchange reaction. **b**, **c** TEM images of hierarchical In_2_S_3_ nanotubes. **d** Elemental mappings of an individual In_2_S_3_ nanotube. **e–g** TEM images of hierarchical In_2_S_3_–CdIn_2_S_4_-10 nanotubes. **h** Elemental mappings of a single In_2_S_3_–CdIn_2_S_4_-10 nanotube. **i–n** CO_2_ photoreduction performance. **i** Generation of CO and H_2_ over different samples. **j** Evolution of CO and H_2_ under various reaction conditions. **k** Results of GC–MS analysis of CO produced from ^13^CO_2_ isotope experiment. **l** Production of CO and H_2_ as a function of reaction time. **m** Formation of CO and H_2_ in stability tests. **n** Wavelength dependence of yields of CO and H_2_, and the light absorption spectrum of In_2_S_3_–CdIn_2_S_4_-10 photocatalyst. Reprinted with the permission from Ref. [74]

Ye’s group developed a MIL-101-derived Fe@C catalyst, consisting of an iron core (< 10 nm) and ultrathin (1–3 layers) carbon layers, for the photocatalytic reduction of CO_2_ to CO [108]. The obtained Fe@C photocatalysts could produce 2196.17 µmol CO under broadband light irradiation for 120 min, which was better than that reported for most of the catalysts. Through a thorough investigation, they found that the intense adsorption of visible light and infrared radiation induced a thermal effect, which helped to drive the reaction, and UV-light-induced iron local surface-plasmon resonances also played a significant role in activating the nonpolar CO_2_ molecules. Furthermore, DFT calculations revealed that the ultrathin layers of carbon shells on the Fe nanoparticles dramatically promoted desorption of the produced CO from the catalyst surface, thereby increasing the CO selectivity (99.76%). Another study on the photocatalytic CO_2_ reduction to CO with MOF-derived materials as catalysts was reported by Lou’s group; they rationally fabricated hierarchical In_2_S_3_–CdIn_2_S_4_ heterostructured nanotubes via sequential ion exchange reactions with MIL-68 as the initial precursor (Fig. 9) [74]. Benefiting from the unique structural and compositional features, such as nanosized interfacial contacts between In_2_S_3_ and CdIn_2_S_4_ nanospecies, reduced diffusion length for charge carrier separation and migration, large surface area for CO_2_ adsorption and concentration, and rich catalytically active sites for surface redox reactions, the obtained hierarchical In_2_S_3_–CdIn_2_S_4_ nanotubes manifested an optimized CO generation rate under visible light. Very recently, a three-component heterojunction C–Cu_2−*x*_S@g-C_3_N_4_ photocatalyst was found to be active toward the reduction of CO_2_ to CO. With MOF-199 as the precursor, hollow tubular Cu_2−*x*_S with carbon coating was successfully fabricated, and further, different amounts of the material were loaded on g-C_3_N_4_ to form a series of C–Cu_2−*x*_S@g-C_3_N_4_ photocatalysts. Under visible-light irradiation and water vapor condition, the optimized C–Cu_2−*x*_S@g-C_3_N_4_ with 0.71 wt% of C–Cu_2−*x*_S exhibited a high reactivity of 1062.6 μmol g^−1^ and selectivity of 97% [109].

Photocatalytic reduction of CO_2_ to hydrocarbons by water vapor with proper catalysts has also been taken into account. Fischer and coworkers have fabricated an Au NP/TiO_2_ (GNP/TiO_2_) photocatalyst active for the reduction of CO_2_ to CH_4_ [110]. After deposition of preformed and surfactant-stabilized gold nanoparticles on the surface of NH_2_-MIL-125, GNP/NH_2_-MIL-125 was transformed to GNP/TiO_2_ through a thermal treatment; the product contained rutile TiO_2_ and possessed the morphology of NH_2_-MIL-125. Compared with the TiO_2_ sample obtained via the pyrolysis of NH_2_-MIL-125 without GNP, P25, and commercial Au/TiO_2_, the GNP/TiO_2_ photocatalyst delivered a significantly higher yield of CH_4_ due to the presence of GNP. Metal oxide composites obtained with MOFs as precursors were also utilized as catalysts for the photocatalytic reduction of CO_2_ to hydrocarbons by Zhang’s group [64]. They fabricated a series of ZnO/NiO porous hollow spheres with sheet-like subunits through the calcination of bimetallic Zn/Ni–MOFs with different ratios of Zn/Ni. The p–n heterojunctions formed by p-type ZnO and n-type NiO, and the hollow character enhanced the performance of ZnO/NiO composites by facilitating the separation of the photogenerated hole–electron carriers. As a result, the optimized ZnO/NiO composites (ZN-30) acted three times better than pure ZnO in the photocatalytic reduction of CO_2_ to CH_3_OH.

To date, despite the high potential in sustainability and energy storage, only few studies have been focused on the photocatalytic reduction of CO_2_ with MOF-derived materials as catalysts. Therefore, more progress should be made on developing more efficient photocatalysts that can enhance the utilization of solar energy and perform better, in terms of both the activity and selectivity.

## Photocatalytic Organic Reaction

The photocatalytic synthesis of organic compounds is a promising approach due to its mild, clean and atom efficiency methodologies, compared with the methods involving high temperature and pressure, and the current industrial synthetic strategies, which generate harmful by-products [112]. Photoinduced charge carriers transferred from semiconductor nanomaterials to organic molecules can catalytically trigger a variety of organic redox reactions. Recently, functionalized MOFs have served as interesting photocatalysts for various organic redox reactions due to their unique, tailorable, and highly porous characteristics. However, the poor thermal stability of MOFs hampers their commercialized utilization [51]. On the other hand, MOF-derived semiconductors, which can inherit the porous structure and diversity in structure from parent MOFs, as photocatalysts, have the advantages of high surface areas, high stability, and controllable band gaps, which can match the different HOMO–LUMO positions of the organic molecules. The photocatalytic mechanism of organic redox reaction shares similar process with other photocatalytic systems, i.e., light adsorption, separation and transfer of photoinduced charge carriers, and the subsequent redox reactions on the surface-active sites of the photocatalysts. In Table 4, some examples of MOF-derived porous structures exhibiting high performance in visible-light-driven photoredox catalysis are given.

**Table 4:** Selected MOF derivatives that serve as photocatalysts for organic reactions

Photocatalyst	MOF precursors	Illumination range	Reaction type	Time (h)	Yield (%)	Recycled times	References
N-doped Cu_2_O@N–C	NTU-105	LED, 450 nm	CDC reaction	24	98.5	5	[63]
CNPC	MOF-199	LED, 450 nm	CDC reaction	25	95	5	[114]
N-doped TiO_2_@N–C	NH_2_-MIL-125	LED, 450 nm	Oxidation coupling of amine	17	99	5	[113]

Long-lived photogenerated carriers play an important role in improving the activity of photocatalysts. Multiple strategies, including structural engineering, semiconductor compositing, doping and so on, have been developed to enhance the transfer and separation efficiency of electrons and holes. The facile method with MOFs as the templates and precursors has been proven ideal for engineering photocatalysts with suitable structures and compositions. Our group have reported N-doped Cu_2_O@N–C NP catalysts derived from a N-rich NTU-105, which share several favorable features for photocatalysis: (1) a porous C matrix substrate, which facilitated uniform distribution of the small Cu_2_O nanoparticles by stabilizing them and preventing their aggregation and (2) high conductivity, attributed to the N-doped Cu_2_O and C substrate, which facilitated electron and hole transfer and separation [63]. The N-doped Cu_2_O@N–C NPs exhibited excellent performance in cross-dehydrogenative coupling (CDC) reactions, owning to the long-lived holes, whose existence was revealed by femtosecond transient absorption spectroscopy. By utilizing NH_2_-MIL-125 as a hard template, N-doped TiO_2_@N–C with butterfly structure (TNPC) was also fabricated as a highly efficient photocatalyst for visible-light-induced amine oxidation [113]. Through an in-depth investigation with femtosecond transient absorption spectroscopy and DFT calculation, the number of actively long-lived holes was found to be in the order TiO_2_ < N-doped TiO_2_ < TNPC, leading to significantly enhanced photocatalytic activity of TNPC.

**Fig. 10:**
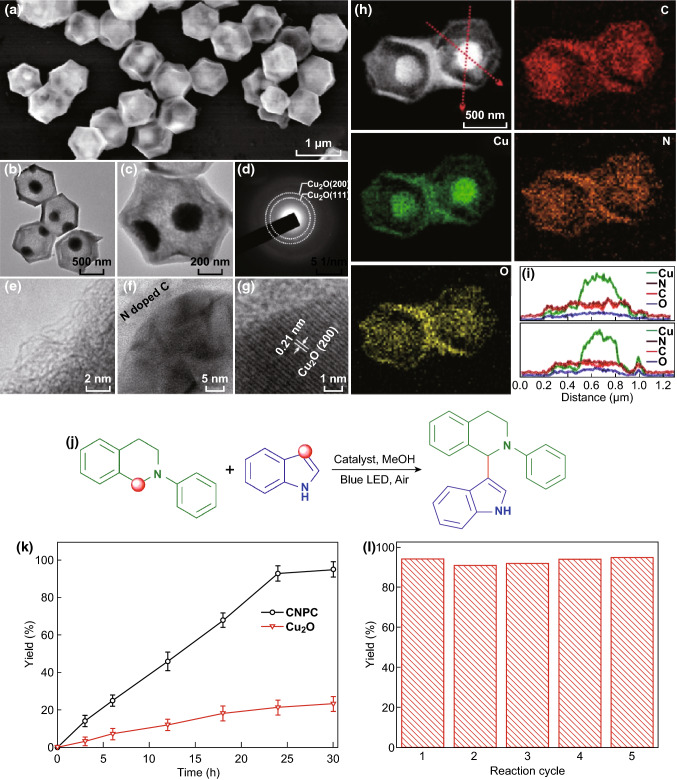
Structure characterization of the core–shell cuboctahedron structure: **a** FESEM image, **b** and **c** TEM images, **d** SAED pattern, and **e–g** the corresponding HRTEM images. **h** STEM image of the core–shell cuboctahedron structure and the corresponding EDXS elemental mapping of C, Cu, N, and O and **i** line profiles. **j** Photocatalytic equation and reaction conditions. **k** Product yield correlation with reaction time in the CDC reaction between indole and *N*-aryl-tetrahydroisoquinoline catalyzed by different catalysts. **l** Cycling experiments using CNPC as the catalyst. Reprinted with the permission from Ref. [114]

Moreover, MOFs have been realized as the templates for the construction of multilevel structures of composites, which can improve the utilization efficiency of light through the multiple reflections, thereby enhancing the activity of photocatalytic organic reactions. Combined with the structural features of N doping and C compositing, Cu_2_O nanoparticles anchored on an N-doped porous carbon yolk–shell cuboctahedral (CNPC) framework were fabricated with a benzimidazole-modified Cu-btc MOF as the precursor, which possessed a multilevel structure at the same time (Fig. 10) [114]. Benefiting from the structural characteristics, the obtained CNPC nanoparticles, as a photocatalyst, provided several favorable features: prolonged lifetime of photogenerated electrons and holes, multiple reflection of light by the yolk–shell structure, and improved stability and dispersibility of Cu_2_O NPs, leading to excellent performance in CDC reactions under visible-light illumination.

In general, research on photocatalytic organic reactions over MOF-derived catalysts is still in its early stage, and more progress, such as development of more efficient photocatalytic systems from MOFs and broadening their applications in photoredox catalysis, needs to be made.

## Photocatalytic Pollutant Degradation

**Fig. 11:**
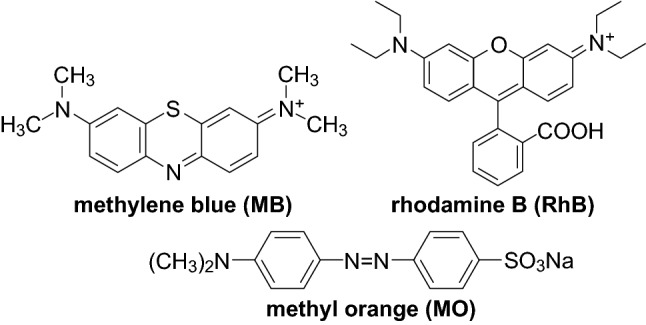
Structures of the three normal dye pollutants

Organic pollutants in water, as an overwhelming problem in environmental chemistry, have raised many concerns. Photodegradation of organic pollutants is considered an ideal strategy to solve the issue, which usually involves O_2_ as the oxidizing agent to degrade organic pollutants to CO_2_, water, and other inorganic species. Among the various photocatalytic systems, MOF-derived materials, such as porous metal oxides, metal sulfides, and their composites (mostly combined with carbon materials) can serve as highly potential photocatalysts due to the merits of high stability, excellent optical absorption/mass transfer, and improved electron–hole separation. In this part, we will summarize the progress in MOF-derived photocatalysts for the degradation of pollutants, which are mostly normal dye pollutants, such as methylene blue (MB), methyl orange (MO), and rhodamine B (RhB) (Fig. 11), and organic pollutants, for example, nitrobenzene. In Table 5, the performances of MOF-derived photocatalysts for pollutant degradation are presented.

**Table 5:** Selected MOF derivatives that serve as photocatalysts for pollutant degradation

Photocatalyst	MOF precursors	*E*_g_ (eV)	Target pollutant	Additive	*k* (min^−1^)	Illumination range	References
ZnO/RGO	ZIF-8–RGO	3.18	MB	None	–	Visible light	[115]
Fe_3_O_4_@C/Cu	Fe_3_O_4_@HKUST-1	1.75	MB	H_2_O_2_	–	Visible light	[131]
ZnS	Zn–MOF	–	MB	None	0.0223	Visible light	[132]
CdS	Cd–MOF	–	MB	None	0.0238	Visible light	[132]
C,N-doped ZnO	ZIF-8	2.98	MB	None	0.068	UV–Vis light	[96]
In_2_O_3_/Co_3_O_4_@PAL composites	Co–In–MOFs–PAL composites	–	MB	None	–	UV–Vis light	[127]
ZnO/3DGN	ZIF-8–3DGN	–	MB	None	–	UV light	[121]
TiO_*x*_/C	Zn–Ti heterometallic MOF	–	MB	None	0.2224	UV light	[118]
TiO_*x*_/C	MIL-125	–	MB	None	0.0207	UV light	[117]
MgO nanorods	Mg–MOF	–	MB	None	–	UV light	[123]
Porous ZnO	ZIF-8	–	MB	None	–	UV light	[61]
In_2_S_3_	In–MOF	2.07	MB	None	–	UV light	[133]
ZnIn_2_S_4_	Zn–MOF and In–MOF	2.30	MB	None	–	UV light	[133]
CdIn_2_S_4_	Cd–MOF and In–MOF	2.30	MB	None	–	UV light	[133]
porous N-doped Cu_2_O/C	Cu–MOF	2.2	MO	None	–	Visible light	[128]
ZnO@C–N–Co core–shell nanostructure	Hollow Zn–Co–ZIF	–	MO	None	–	UV light	[119]
ZnO@Silica	ZIF-8	–	MO	None	–	UV light	[115]
C-doped ZnO	ZIF-8	2.93	RhB	None	0.0015	Visible light	[62]
CoP/Fe_2_P@mC	Co–Fe–MOF	2.11	RhB	None	–	Visible light	[138]
CuO nanofibers	Cu–MOF	–	RhB	H_2_O_2_	0.01712	Visible light	[124]
Zn_0.95_Co_0.05_–ZIF@Zn_0.95_Co_0.05_O	Zn_0.95_Co_0.05_–ZIF	–	RhB	None	0.41	Visible light	[134]
Au/ZnO	GSH-Au NCs–ZIF-8	3.17	RhB	None	–	Visible light	[67]
Porous N-doped ZnO	Urea and ZIF-8 mixture	–	RhB	None	0.0049	Visible light	[120]
Porous ZnO	MOF-5	–	RhB	None	0.0053	UV light	[139]
ZIF-8@Zn_0.95_Ni_0.05_O	ZIF-8–Ni^2+^	–	RhB	None	0.175	UV light	[136]
γ-Fe_2_O_3_/C	MIL-53(Fe)	–	MG	H_2_O_2_	–	Solar light	[129]
CdS/MPC	ZIF-8	–	Cephalexin	None	0.024	UV–Vis light	[137]
ZIF-NC/g-C_3_N_4_	ZIF-8	–	BPA	HSO_5_^−^	0.05134	Visible light	[135]
A/R TiO_2_	MIL-125	3.07	Nitrobenzene	None	–	UV light	[116]
GdCoO_3_	Gd–Co–MOF	–	OG	None	–	UV light	[126]
			RBBR	None	–	UV light	
			RhB	None	–	UV light	
			RBL	None	–	UV light	

### MOF-Derived Porous Metal Oxides

As the most commonly used metal oxides in photocatalysis, TiO_2_ and ZnO show high activities for pollutant photodegradation. With proper MOFs as the precursors, TiO_2_- and ZnO-based photocatalysts with highly improved performances could be obtained via a simple thermal treatment. For example, MIL-125, one of the earliest reported Ti-containing MOFs, is a good choice to fabricate TiO_2_-based photocatalysts, and according to reports, tunable products could be obtained by changing the calcining atmosphere. Pan’s group achieved cake-like porous TiO_2_ particles with the mixed anatase/rutile (A/R TiO_2_) phase via the pyrolysis of MIL-125 under air atmosphere; the as-prepared materials served as photocatalysts for nitrobenzene degradation [116]. Due to the reduced electron–hole pair recombination, the obtained A/R TiO_2_ showed better catalytic activity compared with pure rutile or anatase TiO_2_. Zhao and coworkers found that a series of TiO_*x*_/C composites could be obtained by the thermal treatment of MIL-125 under an Ar atmosphere; these composites exhibited high catalytic activities toward the photodegradation of MB under UV light [117]. In the series of TiO_*x*_/C composites, the product achieved at 1000 °C acted as the best photocatalyst due to its high surface area, reduced Ti_3_O_5_ composition, and conductive carbon matrix. In addition, Zhao and coworkers also reported that the heterometallic MOF (ZTOF-1) containing Zn^2+^ and Ti^4+^ could be used as the precursor to fabricate TiO_*x*_-based catalysts, whereby Zn could be removed via vaporization at 1000 °C, leading to the TiO_*x*_/C composite [118]. The obtained TiO_*x*_/C contained the extra pores formed in the process of vaporization of Zn, and these were readily accessible to MB, leading to a considerable increase in the photocatalytic activity compared with that of the pyrolytic products obtained below 1000 °C.

**Fig. 12:**
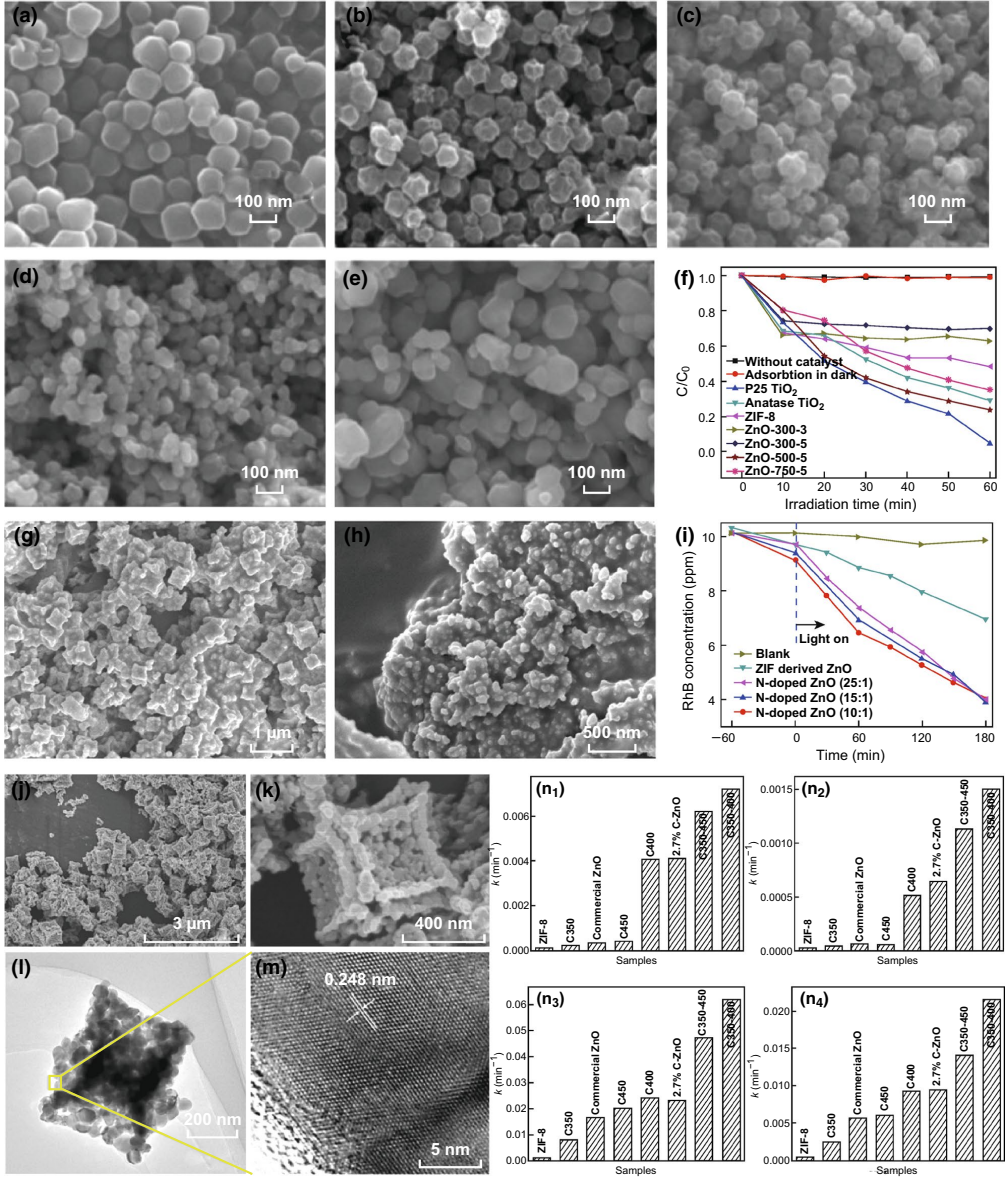
SEM images of **a** ZIF-8, **b** ZnO-300-3, **c** ZnO-300-5, **d** ZnO-500-5, and **e** ZnO-750-5. **f** Photodegradation of MB over different catalysts. SEM images of **g** ZIF-8-derived pure ZnO and **h** N-doped ZnO. **i** Photodegradation of RhB by blank (absence of photocatalyst), pure ZnO, and N-doped ZnO with different ratios of urea and ZIF-8 as the precursors. SEM (**j**, **k**) and TEM (**l**, **m**) images of C350–400. **j** The calculated reaction rates **k** for RhB (*n*_1_) and phenol (*n*_2_) photodegradation under visible light (> 420 nm), *k* for RhB (*n*_3_) and phenol (*n*_4_) photodegradation under UV–Vis light (Xenon light) **a–f** are reprinted with permission from Ref. [61]. **g–i** are reprinted with permission from Ref. [120]. **j–n** are reprinted with permission from Ref. [62]

ZIF-8 or its hybrids have been commonly utilized as the precursors to fabricate ZnO-based materials as catalysts for pollutant photodegradation [67, 115, 119]. In the past few years, various ZnO-based photocatalytic systems, including porous ZnO, porous C- or/and N-doped ZnO, ZnO/carbon materials composites, and other ZnO-based composites, were fabricated (Fig. 12). With pure ZIF-8 as the template and precursor, which contains ligands with nitrogen, i.e., 2-methylimidazole, the resulting products could be tuned as porous ZnO, porous C-doped ZnO, and porous C,N-doped ZnO by changing the calcination conditions, including calcination atmosphere, calcination step, and calcination temperature [61, 62, 96], while, with the mixture of ZIF-8 and urea as the precursors, N-doped ZnO could be obtained [120]. A photocatalytic investigation on these catalysts proved that they all performed well in dye degradation; particularly, porous C-doped ZnO reported by Zhang and coworkers and porous N-doped ZnO reported by Yao’s group exhibited visible-light photodegradation of RhB due to the narrower band gap of C- and N-doped ZnO [62, 120]. When combined with carbon materials such as three-dimensional graphene networks (3DGN), ZIF-8-based hybrids could be transformed to the corresponding ZnO-based hybrids [121]. Zhang’s group developed a method to synthesize the ZnO/3DGN composite via a two-step annealing process with ZIF-8/3DGN as the precursor, whereby ZIF-8/3DGN was fabricated by the direct synthesis of ZIF-8 on 3DGN. The obtained ZnO/3DGN exhibited high activity in MB photodegradation as well as durability. Zhu and coworkers incorporated RGO to ZIF-8-derived ZnO via the microwave-assisted method to fabricate ZnO/RGO hybrids [122]. Attributed to the synergistic effect of enhanced light absorption and suppression of charge carrier recombination resulting from the interaction between ZnO and RGO, the ZnO/RGO composites were able to show higher activity for the visible-light degradation of MB than pure ZnO. In the series of composites with different amounts of RGO, the as-prepared composite with 1.5 wt% of RGO showed an optimal photocatalytic performance.

In addition to TiO_2_ and ZnO, other metal oxide-based photocatalysts for the pollutant degradation have also been derived from the corresponding MOF materials [123–125]. In 2007, Madras and Natarajan et al. reported the photodegradation of four organic pollutants, including RhB, Rhodamine Blue (RBL), Orange G (OG), and Remazol Brilliant Blue R (RBBR), with a bimetallic Gd/Co–MOF-derived nanosized GdCoO_3_ as the catalyst [126]. At different calcination temperatures, GdCoO_3_ with different particle sizes could be obtained. Compared with P25, the as-synthesized ~ 3 nm GdCoO_3_ particles showed higher rates of degradation rates for the four pollutants. Wang’s group has fabricated In_2_O_3_/Co_3_O_4_–palygorskite (PAL) composite photocatalysts for the degradation of MB and tetracycline (TC) from a Co/In–MOF/PAL hybrid [127]. In the presence of PAL, the photocatalytic activities were improved due to their higher adsorption of the substrates and the electrostatic interactions between photogenerated charge carriers and negatively charged PAL. Two-dimensional (2D) porous N-doped Cu_2_O/C composites derived from square-shaped Cu–MOF nanoplatelets as a photocatalyst for MO degradation were reported by Ma and coworkers [128]. The high surface area of the annealed Cu_2_O/C nanoplatelets led to a high degradation rate of 2.5 mg min^−1^g^−1^ for MO pollution under visible light. Moreover, magnetic photocatalysts, such as γ-Fe_2_O_3_/C, Co/graphene materials, Fe_3_O_4_@C/Cu, and Fe_3_O_4_@CuO, for the degradation of pollutants with the advantage of easy separability were fabricated for the degradation of pollutants from the corresponding MOFs or MOF-based composites; these photocatalysts acted well in terms of both activity and reusability [129–131].

### MOF-Derived Porous Metal Sulfides

Various metal sulfides with tunable structures and morphologies have been fabricated via the sulfurization process or thermal treatment of MOFs. For instance, Li and coworkers developed a series of M_*x*_S_y_@C (M = Co, Zn, Cd, Ni, and Cu) through one-pot sulfurization of five MOFs with the identical formula of [M(pa)(bib)]_∞_ [132]. Among them, pure ZnS and CdS were subsequently obtained by the combustion of ZnS@C and CdS@C (to remove carbon) and utilized as moderately active photocatalysts for degradation of MB under visible-light illumination. Batabyal and coworkers developed a one-pot method, i.e., thermolysis of single- or dual-source coordination polymer precursors in the presence of different surfactants, to synthesize nanocrystals of In_2_S_3_, ZnIn_2_S_4_, and CdIn_2_S_4_ [133]. The synthesized In_2_S_3_ nanocrystals showed efficient photocatalytic degradation of MB under UV light irradiation, and the photocatalytic activities of the ternary chalcogenides of In_2_S_3_ were considerably enhanced.

In addition to MOF-derived metal oxides, metal sulfides, and their composites, some other kinds of MOF-derived materials have been reported as highly efficient photocatalysts for the degradation of organic pollutants, such as MOF-derived metal phosphide composites, MOF-derived carbon-modified g-C_3_N_4_, semi-transformed MOFs hybrids, and so on [134–137]. Although there are numerous papers focused on the photodegradation of organic pollutants with porous nanostructures derived from MOFs as photocatalysts, the gap between laboratory research and industrial application is still huge due to the following issues: (1) most of the MOF-derived photocatalysts show limited absorption of visible light and (2) the degradation mechanism is not thoroughly explored, and the degradation products are not clear. Therefore, more in-depth research on MOF-derived photocatalysts for the degradation of organic pollutants should be carried out.

## Summary and Outlook

In this review, we have discussed the improvements in MOF-derived porous structures serving as photocatalysts for various energy-/environment-related reactions, including water splitting, pollutant degradation, CO_2_ reduction, and organic reactions, achieved in the last few years. Owing to the beneficial structural features, such as versatility in components and well-defined pore structures, MOFs have been considered as the ideal precursors of porous semiconductor photocatalysts, including porous metal oxides and porous metal sulfides, and their heterostructures. The derived porous photocatalysts exhibit enhanced performances toward various reactions due to their high accessible surface areas and rich pore structures. Moreover, porous heterostructures or solid solution photocatalysts can be rationally designed from multimetallic MOFs as the precursors. With tuned band gap, increased active sites, and increased efficiency of photogenerated charge carrier separation and migration, the photocatalytic activities of MOF-derived porous heterostructures and solid solutions have been further enhanced.

Despite the great progress achieved in the field of MOF-derived photocatalyst development, there are some pending issues that need to be solved. Transformation processes always involve high-temperature reactions and lack precise control over the pore structure and active sites, which, however, will have great influence on the photocatalytic performances. Therefore, more efforts should be devoted to the controlled synthesis of MOF-derived materials with desired pore structures as well as the active sites, which also call forth in situ techniques to track the transformation process of MOFs. Moreover, MOF-derived semiconductor photocatalysts suffer from low solar-to-chemical-energy conversion efficiency because only a few photocatalytic systems can mainly utilize the energy of the UV region, which is only 5% of the solar spectrum. The development of visible or near-infrared (NIR) light-driven photocatalytic systems with fast kinetics is still in active demand. Fabrication of composites with heterojunctions has shown its superiority in promoting charge separation, yet the intrinsic mechanism is far from well understood. An in-depth investigation should be carried out; the combination of experimental investigation and the corresponding theoretical calculation seems to be an effective method.

Finally, the other issues and their possible recommendations are listed below toward the specific reactions. For photocatalytic water splitting, the currently developed photocatalysts derived from MOFs are aimed at only one isolated half-reaction, and sacrificial reagents are added to replace the other half-reaction. Yet the goal to produce hydrogen at a cost and scale that are comparable with fossil fuels should be realized by rational design of highly efficient water-splitting photocatalysts that can split pure water into stoichiometric amounts of H_2_ and O_2_, driven by sunlight without using any sacrificial reagents. Thus, MOFs-derived photocatalysts for overall water splitting are highly desired. For photocatalytic CO_2_ reduction, the study is in its infancy, and the lower efficiency and selectivity of MOF-derived photocatalysts make it difficult to meet the demands of industrial applications, and therefore, further efforts are needed to make a breakthrough in the fabrication of photocatalysts. From the viewpoints of design principles, the increased adsorption of CO_2_ can improve the reduction efficiency of CO_2_. The exposure of special surface sites that can decrease the energy barrier of the reduction process or the enrichment of CO_2_ molecules around the active sites can help to improve the photocatalytic performance. For photocatalytic organic redox reaction, hole scavengers or electron scavengers are usually used, and designing appropriate tandem reactions can help to avoid their use. With regard to photocatalytic pollutant degradation, research is mainly focused on the degradation of the dye pollutants and some organic pollutants by photogenerated reactive transient species of O_2_. Owing to the non-selective property of the reactive species, the conclusion about the activity of photocatalysts is not accurate. In-depth research on MOF-derived photocatalysts with thoroughly explored degradation mechanism and clear degradation products should be carried out.

In this review, we give a comprehensive survey of MOF-derived porous semiconductor structures as very promising photocatalysts toward various reactions. Given the beneficial features of porous structure and well-defined heterostructures, we believe that MOF-derived porous semiconductor structures will present a bright future toward photocatalysis.

## References

[1] Hoffmann MR, Martin ST, Choi WY, Bahnemann DW (1995). Environmental applications of semiconductor photocatalysis. Chem. Rev..

[2] Fox MA, Dulay MT (1993). Heterogeneous photocatalysis. Chem. Rev..

[3] Mills A, LeHunte S (1997). An overview of semiconductor photocatalysis. J. Photochem. Photobiol., A.

[4] Asahi R, Morikawa T, Ohwaki T, Aoki K, Taga Y (2001). Visible-light photocatalysis in nitrogen-doped titanium oxides. Science.

[5] Sakthivel S, Kisch H (2003). Daylight photocatalysis by carbon-modified titanium dioxide. Angew. Chem. Int. Ed..

[6] Tong H, Ouyang S, Bi Y, Umezawa N, Oshikiri M, Ye J (2012). Nano-photocatalytic materials: possibilities and challenges. Adv. Mater..

[7] Linic S, Christopher P, Ingram DB (2011). Plasmonic-metal nanostructures for efficient conversion of solar to chemical energy. Nat. Mater..

[8] Ni M, Leung MKH, Leung DYC, Sumathy K (2007). A review and recent developments in photocatalytic water-splitting using TiO_2_ for hydrogen production. Renew. Sustain. Energy Rev..

[9] Tu W, Zhou Y, Zou Z (2014). Photocatalytic conversion of CO_2_ into renewable hydrocarbon fuels: state-of-the-art accomplishment, challenges, and prospects. Adv. Mater..

[10] Marschall R (2014). Semiconductor composites: strategies for enhancing charge carrier separation to improve photocatalytic activity. Adv. Funct. Mater..

[11] Maeda K, Domen K (2010). Photocatalytic water splitting: recent progress and future challenges. J. Phys. Chem. Lett..

[12] Du C-F, Liang Q, Dangol R, Zhao J, Ren H, Madhavi S, Yan Q (2018). Layered trichalcogenidophosphate: a new catalyst family for water splitting. Nano Micro Lett..

[13] Su T, Shao Q, Qin Z, Guo Z, Wu Z (2018). Role of interfaces in two-dimensional photocatalyst for water splitting. ACS Catal..

[14] Ong CB, Ng LY, Mohammad AW (2018). A review of ZnO nanoparticles as solar photocatalysts: synthesis, mechanisms and applications. Renew. Sustain. Energy Rev..

[15] Kumar SG, Rao KSRK (2015). Zinc oxide based photocatalysis: tailoring surface-bulk structure and related interfacial charge carrier dynamics for better environmental applications. RSC Adv..

[16] Lee KM, Lai CW, Ngai KS, Juan JC (2016). Recent developments of zinc oxide based photocatalyst in water treatment technology: a review. Water Res..

[17] Rahmanian E, Malekfar R, Pumera M (2018). Nanohybrids of two-dimensional transition-metal dichalcogenides and titanium dioxide for photocatalytic applications. Chem. Eur. J..

[18] Chen B, Meng Y, Sha J, Zhong C, Hua W, Zhao N (2018). Preparation of MoS_2_/TiO_2_ based nanocomposites for photocatalysis and rechargeable batteries: progress, challenges, and perspective. Nanoscale.

[19] Chen X, Liu L, Yu PY, Mao SS (2011). Increasing solar absorption for photocatalysis with black hydrogenated titanium dioxide nanocrystals. Science.

[20] Khan SUM, Al-Shahry M, Ingler WB (2002). Efficient photochemical water splitting by a chemically modified n-TiO_2_. Science.

[21] Ge M, Li Q, Cao C, Huang J, Li S (2017). One-dimensional TiO_2_ nanotube photocatalysts for solar water splitting. Adv. Sci..

[22] Song Y, Li N, Chen D, Xu Q, Li H, He J, Lu J (2018). 3D ordered mop inverse opals deposited with CdS quantum dots for enhanced visible light photocatalytic activity. Appl. Catal. B Environ..

[23] Li Q, Guo B, Yu J, Ran J, Zhang B, Yan H, Gong JR (2011). Highly efficient visible-light-driven photocatalytic hydrogen production of CdS-cluster-decorated graphene nanosheets. J. Am. Chem. Soc..

[24] Zong X, Yan H, Wu G, Ma G, Wen F, Wang L, Li C (2008). Enhancement of photocatalytic H_2_ evolution on CdS by loading MoS_2_ as cocatalyst under visible light irradiation. J. Am. Chem. Soc..

[25] Jiang L, Yuan X, Pan Y, Liang J, Zeng G, Wu Z, Wang H (2017). Doping of graphitic carbon nitride for photocatalysis: a review. Appl. Catal. B Environ..

[26] Zhang G, Lan Z-A, Wang X (2017). Surface engineering of graphitic carbon nitride polymers with cocatalysts for photocatalytic overall water splitting. Chem. Sci..

[27] Fei J, Li J (2015). Controlled preparation of porous TiO_2_–Ag nanostructures through supramolecular assembly for plasmon-enhanced photocatalysis. Adv. Mater..

[28] Sun MH, Huang SZ, Chen LH, Li Y, Yang XY, Yuan ZY, Su BL (2016). Applications of hierarchically structured porous materials from energy storage and conversion, catalysis, photocatalysis, adsorption, separation, and sensing to biomedicine. Chem. Soc. Rev..

[29] Lu B, Li X, Wang T, Xie E, Xu Z (2013). WO_3_ nanoparticles decorated on both sidewalls of highly porous TiO_2_ nanotubes to improve UV and visible-light photocatalysis. J. Mater. Chem. A.

[30] Wang S, Wang X (2015). Multifunctional metal–organic frameworks for photocatalysis. Small.

[31] Qiu B, Xing M, Zhang J (2014). Mesoporous TiO_2_ nanocrystals grown in situ on graphene aerogels for high photocatalysis and lithium-ion batteries. J. Am. Chem. Soc..

[32] Yu J, Su Y, Cheng B (2007). Template-free fabrication and enhanced photocatalytic activity of hierarchical macro-/mesoporous titania. Adv. Funct. Mater..

[33] Liang Q, Li Z, Yu X, Huang ZH, Kang F, Yang QH (2015). Macroscopic 3D porous graphitic carbon nitride monolith for enhanced photocatalytic hydrogen evolution. Adv. Mater..

[34] Chen C, Cai W, Long M, Zhou B, Wu Y, Wu D, Feng Y (2010). Synthesis of visible-light responsive graphene oxide/TiO_2_ composites with p/n heterojunction. ACS Nano.

[35] Wang H, Zhang L, Chen Z, Hu J, Li S, Wang Z, Liu J, Wang X (2014). Semiconductor heterojunction photocatalysts: design, construction, and photocatalytic performances. Chem. Soc. Rev..

[36] Bessekhouad Y, Robert D, Weber JV (2005). Photocatalytic activity of Cu_2_O/TiO_2_, Bi_2_O_3_/TiO_2_ and ZnMn_2_O_4_/TiO_2_ heterojunctions. Catal. Today.

[37] Dong F, Zhao Z, Xiong T, Ni Z, Zhang W, Sun Y, Ho WK (2013). In situ construction of g-C_3_N_4_/g-C_3_N_4_ metal-free heterojunction for enhanced visible-light photocatalysis. ACS Appl. Mater. Interfaces..

[38] Lin D, Wu H, Zhang R, Pan W (2009). Enhanced photocatalysis of electrospun Ag-ZnO heterostructured nanofibers. Chem. Mater..

[39] Low J, Yu J, Jaroniec M, Wageh S, Al-Ghamdi AA (2017). Heterojunction photocatalysts. Adv. Mater..

[40] Zhang Z, Shao C, Li X, Wang C, Zhang M, Liu Y (2010). Electrospun nanofibers of p-type NiO/n-type ZnO heterojunctions with enhanced photocatalytic activity. ACS Appl. Mater. Interfaces..

[41] Sarkar D, Ghosh CK, Mukherjee S, Chattopadhyay KK (2013). Three dimensional Ag_2_O/TiO_2_ type-II (p–n) nanoheterojunctions for superior photocatalytic activity. ACS Appl. Mater. Interfaces..

[42] Cho Y, Kim S, Park B, Lee CL, Kim JK (2018). Multiple heterojunction in single titanium dioxide nanoparticles for novel metal-free photocatalysis. Nano Lett..

[43] Schneemann A, Bon V, Schwedler I, Senkovska I, Kaskel S, Fischer RA (2014). Flexible metal–organic frameworks. Chem. Soc. Rev..

[44] Lohe MR, Gedrich K, Freudenberg T, Kockrick E, Dellmann T, Kaskel S (2011). Heating and separation using nanomagnet-functionalized metal–organic frameworks. Chem. Commun..

[45] Zhu X, Zheng H, Wei X, Lin Z, Guo L, Qiu B, Chen G (2013). Metal–organic framework (MOF): a novel sensing platform for biomolecules. Chem. Commun..

[46] Li S, Huo F (2015). Metal–organic framework composites: from fundamentals to applications. Nanoscale.

[47] Kreno LE, Leong K, Farha OK, Allendorf M, Van Duyne RP, Hupp JT (2012). Metal-organic framework materials as chemical sensors. Chem. Rev..

[48] Furukawa H, Ko N, Go YB, Aratani N, Choi SB (2010). Ultrahigh porosity in metal–organic frameworks. Science.

[49] Maurin G, Serre C, Cooper A, Férey G (2017). The new age of MOFs and of their porous-related solids. Chem. Soc. Rev..

[50] Li Y, Xu H, Ouyang S, Ye J (2016). Metal–organic frameworks for photocatalysis. Phys. Chem. Chem. Phys..

[51] Subudhi S, Rath D, Parida KM (2018). A mechanistic approach towards the photocatalytic organic transformations over functionalised metal organic frameworks: a review. Catal. Sci. Technol..

[52] Dhakshinamoorthy A, Li Z, Garcia H (2018). Catalysis and photocatalysis by metal organic frameworks. Chem. Soc. Rev..

[53] Wu Z, Yuan X, Zhang J, Wang H, Jiang L, Zeng G (2017). Photocatalytic decontamination of wastewater containing organic dyes by metal–organic frameworks and their derivatives. ChemCatChem.

[54] Jiang HL, Liu B, Lan YQ, Kuratani K, Akita T, Shioyama H, Zong FQ, Xu Q (2011). From metal–organic framework to nanoporous carbon: toward a very high surface area and hydrogen uptake. J. Am. Chem. Soc..

[55] Ma X, Zhou YX, Liu H, Li Y, Jiang HL (2016). A MOF-derived Co-CoO@N-doped porous carbon for efficient tandem catalysis: dehydrogenation of ammonia borane and hydrogenation of nitro compounds. Chem. Commun..

[56] Ma B, Guan PY, Li QY, Zhang M, Zang SQ (2016). MOF-derived flower-like MoS_2_@TiO_2_ nanohybrids with enhanced activity for hydrogen evolution. ACS Appl. Mater. Interfaces..

[57] Han X, Chen WM, Han X, Tan YZ, Sun D (2016). Nitrogen-rich MOF derived porous Co_3_O_4_/N-C composites with superior performance in lithium-ion batteries. J. Mater. Chem. A.

[58] Zhao X, Yang H, Jing P, Shi W, Yang G, Cheng P (2017). A metal–organic framework approach toward highly nitrogen-doped graphitic carbon as a metal-free photocatalyst for hydrogen evolution. Small.

[59] Zhang L, Wu HB, Lou XW (2013). Metal–organic-frameworks-derived general formation of hollow structures with high complexity. J. Am. Chem. Soc..

[60] Xia W, Mahmood A, Zou R, Xu Q (2015). Metal–organic frameworks and their derived nanostructures for electrochemical energy storage and conversion. Energy Environ. Sci..

[61] Du Y, Chen RZ, Yao JF, Wang HT (2013). Facile fabrication of porous ZnO by thermal treatment of zeolitic imidazolate framework-8 and its photocatalytic activity. J. Alloys Compd..

[62] Pan L, Muhammad T, Ma L, Huang ZF, Wang S, Wang L, Zou JJ, Zhang X (2016). MOF-derived C-doped ZnO prepared via a two-step calcination for efficient photocatalysis. Appl. Catal. B Environ..

[63] Han X, He X, Wang F, Chen J, Xu J, Wang X, Han X (2017). Engineering an N-doped Cu_2_O@N–C interface with long-lived photo-generated carriers for efficient photoredox catalysts. J. Mater. Chem. A.

[64] Chen J, Yu J, Zhang J (2018). Enhanced photocatalytic CO_2_ reduction activity of MOF-derived ZnO/NiO porous hollow spheres. J. CO2 Util..

[65] Lan M, Guo RM, Dou Y, Zhou J, Zhou A, Li JR (2017). Fabrication of porous Pt-doping heterojunctions by using bimetallic MOF template for photocatalytic hydrogen generation. Nano Energy.

[66] deKrafft KE, Wang C, Lin W (2012). Metal–organic framework templated synthesis of Fe_2_O_3_/TiO_2_ nanocomposite for hydrogen production. Adv. Mater..

[67] He L, Li L, Wang T, Gao H, Li G, Wu X, Su Z, Wang C (2014). Fabrication of Au/ZnO nanoparticles derived from ZIF-8 with visible light photocatalytic hydrogen production and degradation dye activities. Dalton Trans..

[68] Zhang Y, Huang J, Ding Y (2016). Porous Co_3_O_4_/CuO hollow polyhedral nanocages derived from metal–organic frameworks with heterojunctions as efficient photocatalytic water oxidation catalysts. Appl. Catal. B Environ..

[69] Su Y, Ao D, Liu H, Wang Y (2017). MOF-derived yolk–shell CdS microcubes with enhanced visible-light photocatalytic activity and stability for hydrogen evolution. J. Mater. Chem. A.

[70] Kumar DP, Park H, Kim EH, Hong S, Gopannagari M, Reddy DA, Kim TK (2018). Noble metal-free metal–organic framework-derived onion slice-type hollow cobalt sulfide nanostructures: enhanced activity of CdS for improving photocatalytic hydrogen production. Appl. Catal. B: Environ..

[71] Huang ZF, Song J, Li K, Tahir M, Wang YT, Pan L, Wang L, Zhang X, Zou JJ (2016). Hollow cobalt-based bimetallic sulfide polyhedra for efficient all-pH-value electrochemical and photocatalytic hydrogen evolution. J. Am. Chem. Soc..

[72] Zhao X, Feng J, Liu J, Shi W, Yang G, Wang GC, Cheng P (2018). An efficient, visible-light-driven, hydrogen evolution catalyst NiS/Zn_x_Cd_1-x_S nanocrystal derived from a metal–organic framework. Angew. Chem. Int. Ed..

[73] Chen W, Fang J, Zhang Y, Chen G, Zhao S (2018). CdS nanosphere-decorated hollow polyhedral ZCO derived from a metal–organic framework (MOF) for effective photocatalytic water evolution. Nanoscale.

[74] Wang S, Guan BY, Lu Y, Lou XWD (2017). Formation of hierarchical In_2_S_3_–CdIn_2_S_4_ heterostructured nanotubes for efficient and stable visible light CO_2_ reduction. J. Am. Chem. Soc..

[75] Meyer K, Ranocchiari M, van Bokhoven JA (2015). Metal organic frameworks for photo-catalytic water splitting. Energy Environ. Sci..

[76] Wang W, Xu X, Zhou W, Shao Z (2017). Recent progress in metal–organic frameworks for applications in electrocatalytic and photocatalytic water splitting. Adv. Sci..

[77] Yuan YJ, Chen D, Yu ZT, Zou ZG (2018). Cadmium sulfide-based nanomaterials for photocatalytic hydrogen production. J. Mater. Chem. A.

[78] Chen X, Mao SS (2007). Titanium dioxide nanomaterials: synthesis, properties, modifications, and applications. Chem. Rev..

[79] O’Regan B, Grätzel M (1991). A low-cost, high-efficiency solar cell based on dye-sensitized colloidal TiO_2_ films. Nature.

[80] Bala S, Mondal I, Goswami A, Pal U, Mondal R (2014). Synthesis, crystal structure and optical properties of a naphthylbisimide-Ni complex: a framework on TiO_2_ for visible light H_2_ production. Dalton Trans..

[81] Li R, Wu S, Wan X, Xu H, Xiong Y (2016). Cu/TiO_2_ octahedral-shell photocatalysts derived from metal–organic framework@semiconductor hybrid structures. Inorg. Chem. Front..

[82] Minh-Hao P, Cao-Thang D, Gia-Thanh V, Ngoc-Don T, Trong-On D (2014). Visible light induced hydrogen generation using a hollow photocatalyst with two cocatalysts separated on two surface sides. Phys. Chem. Chem. Phys..

[83] Yan B, Zhang L, Tang Z, Al-Mamun M, Zhao H, Su X (2017). Palladium-decorated hierarchical titania constructed from the metal–organic frameworks NH_2_-MIL-125(Ti) as a robust photocatalyst for hydrogen evolution. Appl. Catal. B Environ..

[84] Bala S, Mondal I, Goswami A, Pal U, Mondal R (2015). Co–MOF as a sacrificial template: manifesting a new Co_3_O_4_/TiO_2_ system with a p–n heterojunction for photocatalytic hydrogen evolution. J. Mater. Chem. A.

[85] Yao J, Chen J, Shen K, Li Y (2018). Phase-controllable synthesis of MOF-templated maghemite–carbonaceous composites for efficient photocatalytic hydrogen production. J. Mater. Chem. A.

[86] Li R, Sun L, Zhan W, Li YA, Wang X, Han X (2018). Engineering an effective noble-metal-free photocatalyst for hydrogen evolution: hollow hexagonal porous micro-rods assembled from In_2_O_3_@carbon core–shell nanoparticles. J. Mater. Chem. A.

[87] Xu JY, Zhai XP, Gao LF, Chen P, Zhao M, Yang HB, Cao DF, Wang Q, Zhang HL (2016). In situ preparation of a MOF-derived magnetic carbonaceous catalyst for visible-light-driven hydrogen evolution. RSC Adv..

[88] Xiao JD, Jiang HL (2017). Thermally stable metal–organic framework-templated synthesis of hierarchically porous metal sulfides: enhanced photocatalytic hydrogen production. Small.

[89] Kumar DP, Choi J, Hong S, Reddy DA, Lee S, Kim TK (2016). Rational synthesis of metal–organic framework-derived noble metal-free nickel phosphide nanoparticles as a highly efficient co-catalyst for photocatalytic hydrogen evolution. ACS Sustain. Chem. Eng..

[90] Tang X, Zhao JH, Li YH, Zhou ZJ, Li K, Liu FT, Lan YQ (2017). Co-doped Zn_1−x_Cd_x_S nanocrystals from metal–organic framework precursors: porous microstructure and efficient photocatalytic hydrogen evolution. Dalton Trans..

[91] Chen H, Gu ZG, Mirza S, Zhang SH, Zhang J (2018). Hollow Cu–TiO_2_/C nanospheres derived from a Ti precursor encapsulated MOF coating for efficient photocatalytic hydrogen evolution. J. Mater. Chem. A.

[92] Zhang M, Huang YL, Wang JW, Lu TB (2016). A facile method for the synthesis of a porous cobalt oxide-carbon hybrid as a highly efficient water oxidation catalyst. J. Mater. Chem. A.

[93] Lan Q, Zhang ZM, Qin C, Wang XL, Li YG, Tan HQ, Wang EB (2016). Highly dispersed polyoxometalate-doped porous Co_3_O_4_ water oxidation photocatalysts derived from POM@MOF crystalline materials. Chem. Eur. J..

[94] Feng Y, Wei J, Ding Y (2016). Efficient photochemical, thermal, and electrochemical water oxidation catalyzed by a porous iron-based oxide derived metal organic framework. J. Phys. Chem. C.

[95] Wei J, Feng Y, Liu Y, Ding Y (2015). M_x_Co_3−x_O_4_ (M = Co, Mn, Fe) porous nanocages derived from metal–organic frameworks as efficient water oxidation catalysts. J. Mater. Chem. A.

[96] Liang P, Zhang C, Sun H, Liu S, Tade M, Wang S (2016). Photocatalysis of C,N-doped ZnO derived from ZIF-8 for dye degradation and water oxidation. RSC Adv..

[97] Li B, Li F, Bai S, Wang Z, Sun L, Yang Q, Li C (2012). Oxygen evolution from water oxidation on molecular catalysts confined in the nanocages of mesoporous silicas. Energy Environ. Sci..

[98] Yagi M, Kaneko M (2001). Molecular catalysts for water oxidation. Chem. Rev..

[99] Jiao F, Frei H (2010). Nanostructured cobalt and manganese oxide clusters as efficient water oxidation catalysts. Energy Environ. Sci..

[100] Hong D, Yamada Y, Nagatomi T, Takai Y, Fukuzumi S (2012). Catalysis of nickel ferrite for photocatalytic water oxidation using [Ru(bpy)_3_]^2+^ and S_2_O_8_^2−^. J. Am. Chem. Soc..

[101] Huang J, Hu G, Ding Y, Pang M, Ma B (2016). Mn-doping and NiFe layered double hydroxide coating: effective approaches to enhancing the performance of α-Fe_2_O_3_ in photoelectrochemical water oxidation. J. Catal..

[102] Robinson DM, Go YB, Mui M, Gardner G, Zhang Z (2013). Photochemical water oxidation by crystalline polymorphs of manganese oxides: structural requirements for catalysis. J. Am. Chem. Soc..

[103] Lu F, Zhou M, Zhou Y, Zeng X (2017). First-row transition metal based catalysts for the oxygen evolution reaction under alkaline conditions: basic principles and recent advances. Small.

[104] Zhou M, Weng Q, Popov ZI, Yang Y, Antipina LY, Sorokin PB, Wang X, Bando Y, Golberg D (2018). Construction of polarized carbon–nickel catalytic surfaces for potent, durable, and economic hydrogen evolution reactions. ACS Nano.

[105] Zhou M, Weng Q, Zhang X, Wang X, Xue Y, Zeng X, Bando Y, Golberg D (2017). In situ electrochemical formation of core–shell nickel–iron disulfide and oxyhydroxide heterostructured catalysts for a stable oxygen evolution reaction and the associated mechanisms. J. Mater. Chem. A.

[106] Lu X, Zhao C (2015). Electrodeposition of hierarchically structured three-dimensional nickel–iron electrodes for efficient oxygen evolution at high current densities. Nat. Commun..

[107] Li R, Zhang W, Zhou K (2018). Metal–organic-framework-based catalysts for photoreduction of CO_2_. Adv. Mater..

[108] Zhang H, Wang T, Wang J, Liu H, Dao TD (2016). Surface-plasmon-enhanced photodriven CO_2_ reduction catalyzed by metal–organic-framework-derived iron nanoparticles encapsulated by ultrathin carbon layers. Adv. Mater..

[109] Hu CY, Zhou J, Sun CY, Chen MM, Wang XL, Su ZM (2018). HKUST-1 derived hollow C–Cu_2−x_S nanotube/g-C_3_N_4_ composites for visible-light CO_2_ photoreduction with H_2_O vapor. Chemistry.

[110] Khaletskaya K, Pougin A, Medishetty R, Rosler C, Wiktor C, Strunk J, Fischer RA (2015). Fabrication of gold/titania photocatalyst for CO_2_ reduction based on pyrolytic conversion of the metal–organic framework NH_2_-MIL-125(Ti) loaded with gold nanoparticles. Chem. Mater..

[111] Yan S, Yu Y, Cao Y (2019). Synthesis of porous ZnMn_2_O_4_ flower-like microspheres by using MOF as precursors and its application on photoreduction of CO_2_ into CO. Appl. Surf. Sci..

[112] Narayanam JMR, Stephenson CRJ (2011). Visible light photoredox catalysis: applications in organic synthesis. Chem. Soc. Rev..

[113] Wang F, He X, Sun L, Chen J, Wang X, Xu J, Han X (2018). Engineering an N-doped TiO_2_@N-doped C butterfly-like nanostructure with long-lived photo-generated carriers for efficient photocatalytic selective amine oxidation. J. Mater. Chem. A.

[114] Han X, He X, Sun L, Han X, Zhan W, Xu J, Wang X, Chen J (2018). Increasing effectiveness of photogenerated carriers by in situ anchoring of Cu_2_O nanoparticles on a nitrogen-doped porous carbon yolk–shell cuboctahedral framework. ACS Catal..

[115] Ahmed A, Forster M, Jin J, Myers P, Zhang H (2015). Tuning morphology of nanostructured ZIF-8 on silica microspheres and applications in liquid chromatography and dye degradation. ACS Appl. Mater. Interfaces..

[116] Li J, Xu X, Liu X, Qin W, Wang M, Pan L (2017). Metal-organic frameworks derived cake-like anatase/rutile mixed phase TiO_2_ for highly efficient photocatalysis. J. Alloys Compd..

[117] Guo Z, Cheng JK, Hu Z, Zhang M, Xu Q, Kang Z, Zhao D (2014). Metal–organic frameworks (MOFs) as precursors towards TiO_x_/C composites for photodegradation of organic dye. RSC Adv..

[118] Xu Q, Guo Z, Zhang M, Hu Z, Qian Y, Zhao D (2016). Highly efficient photocatalysts by pyrolyzing a Zn–Ti heterometallic metal–organic framework. CrystEngComm.

[119] Chen H, Shen K, Chen J, Chen X, Li Y (2017). Hollow–ZIF-templated formation of a ZnO@C–N–Co core–shell nanostructure for highly efficient pollutant photodegradation. J. Mater. Chem. A.

[120] Feng Y, Lu H, Gu X, Qiu J, Jia M, Huang C, Yao J (2017). ZIF-8 derived porous N-doped ZnO with enhanced visible light-driven photocatalytic activity. J. Phys. Chem. Solids.

[121] Cao X, Zheng B, Rui X, Shi W, Yan Q, Zhang H (2014). Metal oxide-coated three-dimensional graphene prepared by the use of metal–organic frameworks as precursors. Angew. Chem. Int. Ed..

[122] Zhu G, Li X, Wang H, Zhang L (2017). Microwave assisted synthesis of reduced graphene oxide incorporated MOF-derived ZnO composites for photocatalytic application. Catal. Commun..

[123] Salehifar N, Zarghami Z, Ramezani M (2016). A facile, novel and low-temperature synthesis of MgO nanorods via thermal decomposition using new starting reagent and its photocatalytic activity evaluation. Mater. Lett..

[124] Zeng QX, Xu GC, Zhang L, Lin H, Lv Y, Jia DZ (2018). Porous CuO nanofibers derived from a Cu-based coordination polymer as a photocatalyst for the degradation of rhodamine B. New J. Chem..

[125] Aly HM, Moustafa ME, Nassar MY, Abdelrahman EA (2015). Synthesis and characterization of novel Cu(II) complexes with 3-substituted-4-amino-5-mercapto-1,2,4-triazole Schiff bases: a new route to CuO nanoparticles. J. Mol. Struct..

[126] Mahata P, Aarthi T, Madras G, Natarajan S (2007). Photocatalytic degradation of dyes and organics with nanosized GdCoO_3_. J. Phys. Chem. C.

[127] Xu J, Gao J, Liu Y, Li Q, Wang L (2017). Fabrication of In_2_O_3_/Co_3_O_4_-palygorskite composites by the pyrolysis of In/Co–MOFs for efficient degradation of methylene blue and tetracycline. Mater. Res. Bull..

[128] Lin Y, Wan H, Chen F, Liu X, Ma R, Sasaki T (2018). Two-dimensional porous cuprous oxide nanoplatelets derived from metal–organic frameworks (MOFs) for efficient photocatalytic dye degradation under visible light. Dalton Trans..

[129] Zhang C, Ye F, Shen S, Xiong Y, Su L, Zhao S (2015). From metal–organic frameworks to magnetic nanostructured porous carbon composites: towards highly efficient dye removal and degradation. RSC Adv..

[130] Andrew Lin KY, Hsu FK, Lee WD (2015). Magnetic cobalt–graphene nanocomposite derived from self-assembly of MOFs with graphene oxide as an activator for peroxymonosulfate. J. Mater. Chem. A.

[131] Zhang YF, Qiu LG, Yuan YP, Zhu YJ, Jiang X, Xiao JD (2014). Magnetic Fe_3_O_4_@C/Cu and Fe_3_O_4_@CuO core–shell composites constructed from MOF-based materials and their photocatalytic properties under visible light. Appl. Catal. B Environ..

[132] Li ZX, Yang BL, Jiang YF, Yu CY, Zhang L (2018). Metal-directed assembly of five 4-connected MOFs: one-pot syntheses of MOF-derived M_x_S_y_@C composites for photocatalytic degradation and supercapacitors. Cryst. Growth Des..

[133] Batabyal SK, Lu SE, Vittal JJ (2016). Synthesis, characterization, and photocatalytic properties of In_2_S_3_, ZnIn_2_S_4_, and CdIn_2_S_4_ nanocrystals. Cryst. Growth Des..

[134] Yang X, Chen J, Hu J, Zhao S, Zhao J, Luo X (2018). Metal organic framework-derived Zn_1−x_Co_x_–ZIF@Zn_1−x_Co_x_O hybrid photocatalyst with enhanced photocatalytic activity through synergistic effect. Catal. Sci. Technol..

[135] Gong Y, Zhao X, Zhang H, Yang B, Xiao K (2018). MOF-derived nitrogen doped carbon modified g-C_3_N_4_ heterostructure composite with enhanced photocatalytic activity for bisphenol a degradation with peroxymonosulfate under visible light irradiation. Appl. Catal. B Environ..

[136] Jing Y, Wang J, Yu B, Lun J, Cheng Y (2017). A MOF-derived ZIF-8@Zn_1−x_Ni_x_O photocatalyst with enhanced photocatalytic activity. RSC Adv..

[137] Yang C, Cheng J, Chen Y, Hu Y (2017). CdS nanoparticles immobilized on porous carbon polyhedrons derived from a metal–organic framework with enhanced visible light photocatalytic activity for antibiotic degradation. Appl. Surf. Sci..

[138] Hu B, Yuan JY, Tian JY, Wang M, Wang X, He L, Zhang Z, Wang ZW, Liu CS (2018). Co/Fe-bimetallic organic framework-derived carbon-incorporated cobalt–ferric mixed metal phosphide as a highly efficient photocatalyst under visible light. J. Colloid Interface Sci..

[139] Yang SJ, Im JH, Kim T, Lee K, Park CR (2011). MOF-derived ZnO and ZnO@C composites with high photocatalytic activity and adsorption capacity. J. Hazard. Mater..

